# A Glycopolymer Sensor Array That Differentiates Lectins and Bacteria

**DOI:** 10.1021/acs.biomac.4c01129

**Published:** 2024-10-18

**Authors:** Kathryn G. Leslie, Katrina A. Jolliffe, Markus Müllner, Elizabeth J. New, W. Bruce Turnbull, Martin A. Fascione, Ville-Petri Friman, Clare S. Mahon

**Affiliations:** a Department of Chemistry, Durham University, Durham DH1 3LE, U.K.; b School of Chemistry University of Sydney, Sydney, NSW 2006, Australia; c Australian Research Council Centre of Excellence for Innovations in Peptide and Protein Science, University of Sydney, Sydney, NSW 2006, Australia; d Key Centre for Polymers and Colloids, School of Chemistry, University of Sydney, Sydney, NSW 2006, Australia; e Sydney Nano Institute, University of Sydney, Sydney, NSW 2006, Australia; f School of Chemistry and Astbury Centre for Structural Molecular Biology, University of Leeds, Leeds LS2 9JT, U.K.; g Department of Chemistry and York Structural Biology Laboratory, University of York, York YO10 5DD, U.K.; h Department of Biology, University of York, York YO10 5DD, U.K.; i Department of Microbiology, Faculty of Agriculture and Forestry, University of Helsinki, Helsinki FI-00014, Finland; j Viikki Biocenter, University of Helsinki, POB 56, Helsinki FI-00014, Finland

## Abstract

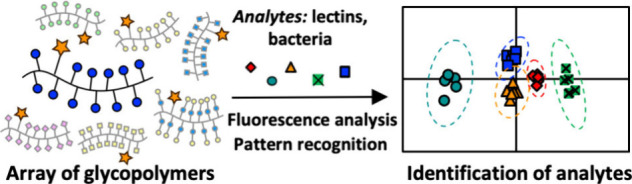

Identification of bacterial lectins offers an attractive route to the development of new diagnostics, but the design of specific sensors is complicated by the low selectivity of carbohydrate–lectin interactions. Here we describe a glycopolymer-based sensor array which can identify a selection of lectins with similar carbohydrate recognition preferences through a pattern-based approach. Receptors were generated using a polymer scaffold functionalized with an environmentally sensitive fluorophore, along with simple carbohydrate motifs. Exposure to lectins induced changes in the emission profiles of the receptors, enabling the discrimination of analytes using linear discriminant analysis. The resultant algorithm was used for lectin identification across a range of concentrations and within complex mixtures of proteins. The sensor array was shown to discriminate different strains of pathogenic bacteria, demonstrating its potential application as a rapid diagnostic tool to characterize bacterial infections and identify bacterial virulence factors such as production of adhesins and antibiotic resistance.

## Introduction

Recognition events between glycans and carbohydrate-binding proteins (lectins) are ubiquitous in biology, underpinning important biological processes as diverse as cellular recognition and immune response.^1^ Often, the recognition of carbohydrates on cellular surfaces is exploited by proteins produced by pathogens to enable key processes of disease such as cellular adhesion or entry,^2^ aided by the multivalent presentation of these recognition motifs. As such, these recognition processes are attractive targets for the development of synthetic receptors or inhibitors,^3^ which present opportunities for the development of new therapeutics or diagnostic tools.^4−7^ Macromolecular architectures such as nanoparticles, dendrimers and other polymers are well-suited to this approach, simultaneously affording convenient access to the large interface areas often implicated in biological recognition^8^ and easily facilitating multivalent ligand incorporation to amplify the effects of comparatively weak interactions between carbohydrates and lectins.^9^ While impressive lectin recognition has been achieved using synthetic glycoconjugates,^10−16^ the use of these receptors as diagnostic tools is typically frustrated by the generally low selectivity of carbohydrate-lectin recognition. Lectins may recognize complex oligosaccharides with high affinities but will also bind to simpler carbohydrate motifs with greatly decreased affinities and selectivities, which can limit the use of simplified receptor structures, particularly within complex biological environments. A well-known example of this effect is the carbohydrate recognition domain of the cholera toxin (CTB), which can recognize the GM1 pentasaccharide motif displayed on cellular surfaces with a *K*_d_ of approximately 40 nM, but will recognize its constituent monosaccharides with *K*_d_’s only in the mM range.^17^ This effect hampers the design of the accessible and commercially viable diagnostics that are needed to identify bacterial infections at point-of-care.

The cross-reactivity in the recognition of lectins by carbohydrates presents, however, an ideal opportunity to apply array-based methods^18−21^ for their identification. Here, rather than developing a specific receptor for the analyte of interest, a selection of receptors of low- to medium- selectivity for a range of similar analytes are used concurrently. Analytes are then identified by their unique “fingerprint” response to the array of sensors. Differential sensor arrays have been used to identify sugars,^22^ drugs,^23,24^ proteins,^25,26^ cell types^27,28^ and some bacterial strains,^29,30^ and have even been demonstrated to function in complex biological media^31^ such as human serum. An array-based approach has been used to successfully discriminate fluorescently labeled lectins with similar carbohydrate-binding preferences by exploiting their adhesion to glycosylated surfaces.^32^ In this work, we demonstrate that an array of eight fluorescent glycopolymers constructed using simple, commercially available sugars can be used to discriminate a model lectin library containing plant- and bacterially derived lectins, as well as different strains of bacteria including common hospital-acquired infections and antibiotic resistant strains. The use of a conserved polymer scaffold offers convenient access to arrays of multivalent receptors, presenting a straightforward route for the generation of glycopolymer-based receptor libraries.

## Experimental Section

### General Experimental Details

All reagents were purchased from Sigma-Aldrich, Combi-Blocks or Carbosynth and used as received unless otherwise stated. *N*,*N*-Dimethylacrylamide was passed through basic Al_2_O_3_ immediately prior to use. Lectins were purchased from Sigma-Aldrich unless otherwise stated. **M1** was prepared according to a previously published procedure,^15^ with details of characterization provided in the Supporting Information. ^1^H and ^13^C NMR spectra were recorded on a Bruker Avance 300 spectrometer at 300 and 75 MHz respectively, or on a Bruker Avance 200 spectrometer with ^1^H at 200 MHz, using the residual solvent signal as an internal standard. Gel permeation chromatography was conducted using a Shimadzu Prominence instrument equipped with a refractive index detector and a pair of Phenogel columns (Phenomenex, 300 mm × 7.8 mm; 5 μm 10^4^ Å and 500 Å) in series, at 50 °C with dimethylacetamide (DMAc) containing butylated hydroxytoluene (BHT) (0.05% w/w) and LiBr (0.03% w/w) as the eluent. Near monodisperse poly(methyl methacrylate) standards were used for calibration. UV–vis spectra were collected using a Shimadzu UV-2450 instrument. Fluorescence analysis was performed using a PerkinElmer EnSpire multimode plate reader, or a Tecan Infinite 200 Pro multimode plate reader.

### Synthesis of Naphthalimide **1**

See Scheme 1.

**Scheme 1:**
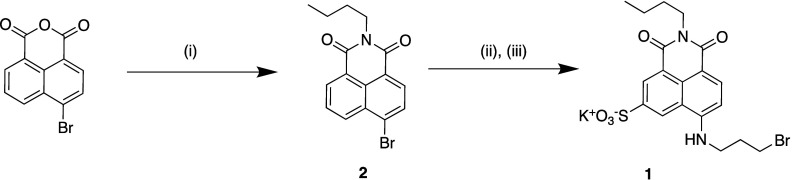
Preparation of **1** (i) C_4_H_9_NH_2_, EtOH, 80 °C, 16 h, (ii) H_2_SO_4_·SO_3_, 90 °C, 3 h, (iii) 3-bromo-propylamine hydrobromide, K_2_CO_3_, CuSO_4_.5H_2_O, EtOH, 80 °C, 48 h.

### *N*-Butyl-4-bromo-1,8-naphthalimide (**2**)

Synthesis was adapted from a published procedure.^33^ Butylamine (0.40 mL, 4.0 mmol) was added to a suspension of 4-bromo-naphthalic anhydride (1.0 g, 3.6 mmol) in EtOH (50 mL), and the mixture was heated under reflux for 16 h. The reaction mixture was poured onto ice (150 mL), and the resulting precipitate was collected by filtration. The crude product was recrystallized from EtOH, then washed with cold water (15 mL) to yield **2** as a pale-yellow solid (0.71 g, 60%). ^1^H NMR (200 MHz, CDCl_3_): δ 8.61 (d, *J* = 7.3, 1H), 8.51 (d, *J* = 8.6, 1H), 8.36 (d, *J* = 7.9, 1H), 8.00 (d, *J* = 7.9, 1H), 7.80 (dd, *J* = 8.6, 7.3, 1H), 4.16 (t, *J* = 7.6, 2H), 1.76–1.66 (m, 2H), 1.50–1.38 (m, 2H), 0.97 (t, *J* = 7.4, 3H); Melting point 106–108 °C, (108–110 °C).^34^

### *N*-Butyl-4-propylbromo-6-sulfo-1,8-naphthalimide (**1**)

*N*-Butyl-4-bromo-1,8-naphthalimide **2** (0.20 g, 0.60 mmol) was added to fuming sulfuric acid (2 mL) at 0 °C. The reaction mixture was warmed to 90 °C under a N_2_ atmosphere and stirred for 20 h. It was then cooled to room temperature and added dropwise to distilled water and ice (50 mL). The mixture was adjusted to pH 8 with NaHCO_3_ solution and the resulting precipitate was collected by filtration, taken up in dioxane and lyophilized. The crude residue was used without further purification. A portion of this material (0.91 g) was dissolved in DMF (10 mL). 3-Bromo-propylamine hydrochloride (0.17 mg, 0.78 mmol), potassium carbonate (0.14 g, 1.0 mmol) and copper(I) chloride (catalytic, approximately 2 mg) were added and the mixture was heated under reflux for 5 h. The solvent was removed under reduced pressure and the crude residue was taken up in CH_2_Cl_2_/MeOH. A white solid was removed by filtration and the filtrate was evaporated to dryness, yielding an orange solid which was purified by flash column chromatography (Teledyne-ISCO CombiFlash, SiO_2_, Rf gold cartridge, hexane → 80:20 hexane:EtOAc) (0.07 g, 25% over two steps). ^1^H NMR (300 MHz, MeOD): δ 8.84 (s, 1H), 8.77 (s, 1H), 8.20 (d, *J* = 8.7, 1H), 6.41 (d, *J* = 8.7, 1H), 4.63–4.50 (m, 4H), 4.14–4.03 (m, 2H), 2.67–2.53 (m, 2H), 1.72–1.59 (m, 2H), 1.50–1.33 (m, 2H), 0.98 (t, *J* = 7.3, 3H); ^13^C NMR (75 MHz, MeOD): δ 165.5, 165.4, 154.5, 141.8, 135.3, 132.2, 129.5, 129.3, 123.5, 120.9, 109.4, 107.7, 56.5, 40.9, 31.3, 21.3, 17.9, 14.2, 9.2; IR ν_max_ 2957, 1635, 1574, 1353, 1193; Melting point: decomp. at 285 °C; HRMS (ESI+) C_19_H_22_N_2_O_5_Br^+^ calculated 469.0433; actual 469.0419.

### BOC-Protected Acylhydrazide Copolymer (**P1**)

*S*-1-Dodecyl-*S’*-(α,α-dimethyl-α”-acetic acid)trithiocarbonate^35^ (DDMAT) (20 mg, 5.5 × 10^–5^ mol, 1.0 equiv), α,α′-azoisobutyronitrile (AIBN) (1.8 mg, 1.1 × 10^–5^ mol, 0.2 equiv), *N,N*-dimethylacrylamide (DMA) (0.462 g, 4.46 mmol, 85 equiv) and **M1** (0.165 g, 8.23 × 10^–4^ mol, 15 equiv) were combined in DMF (4 mL). N_2(g)_ was bubbled through the solution for 15 min, then the vessel was placed in a preheated oil bath at 70 °C. After 18 h the polymerization was quenched by rapid cooling in N_2(l)_ followed by exposure to air. The solution was added dropwise to rapidly stirring Et_2_O, yielding **P1** as a yellow-white solid which was isolated by filtration and dried under high vacuum (0.619 g). ^**1**^H NMR (300 MHz, CDCl_3_): δ 1.0–1.8 (br, CHC*H*_2_), 1.5 (br, COOC(C*H*_3_)_3_), 2.0–2.3 (br, CH_2_C(C*H*_3_)CO), 2.3–2.7 (br, C*H*CH_2_), 2.8–3.2 (br, N(C*H*_3_)_2_).

### Acylhydrazide Functionalized Polymer (**P2**)

**P1** (0.300 g, 2.61 × 10^–5^ mol) was dissolved in CH_2_Cl_2_ (3 mL). Trifluoroacetic acid (3 mL) was added and the solution was left to stir at room temperature for 2 h. The solution was concentrated under a stream of N_2(g)_, yielding a yellow glassy film which was redissolved in H_2_O and lyophilized to afford **P2** as a yellow-white solid (0.225 g, 86% yield). ^1^H NMR (300 MHz, D_2_O) δ 1.0–1.8 (br, CHC*H*_2_), 2.0–2.3 (br, CH_2_C(C*H*_3_)CO), 2.3–2.7 (br, C*H*CH_2_), 2.8–3.2 (br, N(C*H*_3_)_2_).

### Naphthalimide Functionalized Polymer (**P3**)

**P2** (0.100 g, 1.00 × 10^–5^ mol) and *N*-butyl-4-propylbromo-6-sulfo-1,8-naphthalimide **1** (4.7 mg, 1.0 × 10^–5^ mol) were combined in D_2_O (500 μL). After 16 h ^1^H NMR spectroscopic analysis of the solution suggested that the reaction had proceeded to completion. The solution was dialyzed against H_2_O and lyophilized, yielding **P3** as an orange-yellow solid (0.088 g, 84% yield). ^1^H NMR (300 MHz, D_2_O) δ 1.0–1.8 (br, CHC*H*_2_), 2.0–2.3 (br, CH_2_C(C*H*_3_)CO), 2.3–2.7 (br, C*H*CH_2_), 2.8–3.2 (br, N(C*H*_3_)_2_), 6.5 (br, Ar), 8.0 (br, Ar), 8.5 (br, Ar).

### Carbohydrate Decorated Polymers (**P3**-(carbohydrate))

**P3** (40 mg) was dissolved in 100 mM NH_4_OAc pH 4.5 (4 mL). 500 μL aliquots of this solution (5.0 mg, 1.0 equiv) were added to carbohydrates (70 equiv) and the solutions were left at room temperature in the dark for 18 h. Solutions were dialyzed against H_2_O and lyophilized, yielding naphthalimide labeled glycopolymers (4–5 mg, 55–82%).

### Expression and Purification of LTB

Cells from a glycerol stock of *Vibrio sp60* harboring plasmid pMMB68^36^ (kindly provided by Prof. Tim Hirst) were used to inoculate growth medium (100 mL, 25 g/L LB mix, 15 g/L NaCl, ampicillin 100 μg/mL). The culture was grown overnight at 30 °C with shaking at 200 rpm, then used to inoculate fresh growth medium (6 × 1 L, 25 g/L LB mix, 15 g/L NaCl, ampicillin 100 μg/mL). These cultures were incubated at 30 °C with shaking at 200 rpm until A_600_ reached 0.6 before protein expression was induced by addition of isopropyl β-D-1-thiogalactopyranoside to a concentration of 0.5 mM. Cultures were incubated (30 °C, 200 rpm) for a further 24 h, then cells were removed by centrifugation (7500 *g*, 15 min). The combined supernatant was treated with ammonium sulfate (550 g/L) and left to stir at 5 °C for 2 h. Crude protein was isolated by centrifugation (17,000 *g*, 25 min) and redissolved in in 100 mM NaH_2_PO_4_, pH 7.0, 500 mM NaCl (60 mL). Insoluble material was removed by centrifugation (5000 *g*, 10 min) before the solution was passed through a 0.22 μm filter then loaded onto a lactose-sepharose 6B column and eluted with 300 mM lactose, 100 mM NaH_2_PO_4_, pH 7.0, 500 mM NaCl. LTB was dialyzed against PBS (137 mM NaCl, 2.7 mM KCl, 10 mM Na_2_HPO_4_, 1.8 mM KH_2_PO_4_), pH 7.4, lyophilized and stored at −20 °C.

### Isolation of Protein Fractions of Nut Butters

Nut butter (100 mg) was dispersed in 10 mM HEPES, 1 mM CaCl_2_, 1 mM MnCl_2_ pH 7.4 (500 μL) and the suspension was washed with hexane (3 × 500 μL). The insoluble fraction was removed by centrifugation (2000 *g*, 60 s) and the supernatant was passed through a 0.22 μm filter. The presence of protein in the sample was confirmed by a positive test with Bradford reagent (Bio-Rad). UV–vis spectra can be found in the Supporting Information (Figure S2).

### Discrimination of Lectins Using Emission Change Ratio

Solutions of receptors **P3-(carbohydrate)**were prepared at 5.0 μM concentrations in either 10 mM HEPES, 1 mM MnCl_2_, 1 mM CaCl_2_, pH 7.4 for detection of PNA, WGA, SBA and ConA, or 137 mM NaCl, 2.7 mM KCl, 10 mM Na_2_HPO_4_ and 1.8 mM KH_2_PO_4_ for detection of LTB. Solutions were transferred to a 96-well plate (100 μL/well, 6 replicates per receptor) and emission spectra were recorded (λ_ex_ 470 nm, em 500–700 nm). A 10 μL aliquot of lectin analyte (250 μM WGA, SBA, Con A, LTB subunits, 125 μM PNA subunit; each in appropriate buffer) was added to each well. The plate was shaken (3 × 10 s) and left at 21 °C for 15 min before emission spectra were acquired as before. The change in emission in response to lectin addition was calculated:
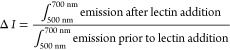


To assess the effects of dilution on emission of receptors, 10 μL aliquot of HEPES buffer was added to each of the receptors (6 replicates). Through visual inspection the effects of dilution were judged to be minimal and similar across all receptors. Linear discriminant analysis (LDA) was performed in SPSS (IBM), with this data set used as training set to construct a scoring model for the identification of unknowns. Raw data and LDA can be found in the Supporting Information, Section 2.1.

### Discrimination of Lectins Using Raw Emission Data

The raw data obtained from emission analysis of solutions containing receptors (**P3-(carbohydrate)**) and lectin analytes (Table S5) was subjected to LDA in SPSS (IBM) to develop the model (Table S6). In this case the effect of nonexposure to analyte was accounted for by the inclusion of the control grouping, where receptors were exposed to an equivalent amount of buffer to that added during lectin addition. LDA enabled effective discrimination of the analytes (Figure S7), with 86.2% of the variance accounted for by the first function. The predictive ability of the array was again assessed through a leave-one-out validation procedure, resulting in identification of analytes with 100% accuracy. Raw data and LDA can be found in the Supporting Information, Section 2.2.

### Identification of Lectins Using Emission Change Ratio

**Figure 1:**
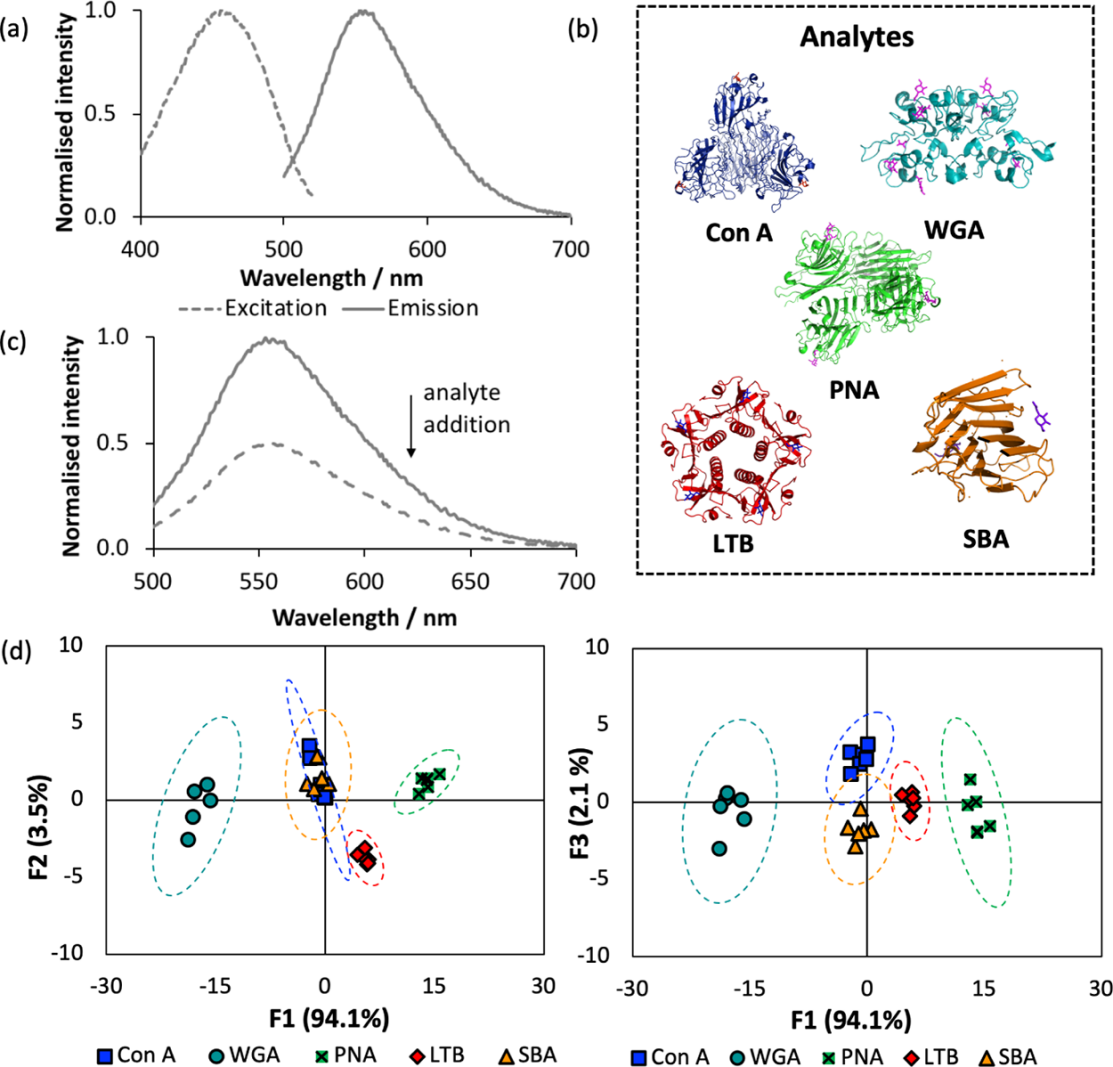
(a) Representative excitation and emission spectra of glycopolymer receptors. (b) Lectin analytes investigated in this study: *E. coli* heat labile toxin (LTB) (1lta.pdb); Concanavalin A (Con A) (5cna.pdb); peanut agglutinin (PNA) (2pel.pdb); wheatgerm agglutinin (WGA) (2uvo.pdb); soybean agglutinin (SBA) (1sbe.pdb). (c) Representative receptor response to analyte exposure (**P3-GlcNAc** 5.0 μM, WGA 250 μM subunit concentration). (d) Canonical LDA score plots for the analysis of lectins performed in sextuplicate (5.0 μM receptors, 125–250 μM lectin subunit, pH 7.4). The pairing of the first (F1) and second (F2) and first and third (F3) factors is shown in separate 2D plots. Dashed lines indicate 95% confidence intervals.

Solutions of receptors were prepared as above. After acquisition of emission spectra as described above, solutions of unknown analytes (10 μL/well, 6 replicates per receptor) were added. The plate was shaken (3 × 10 s) and left at 21 °C for 15 min before emission spectra were acquired as before. The change in emission in response to lectin addition was calculated (Table S9), and unknown analytes were scored using the model constructed (Table S4). Score functions 1 and 2 for each sample were calculated using the discriminant functions obtained for the training set to allow graphical representation (Figure 1d).

### Identification of Lectins within Complex Samples Using Emission Change Ratio

Emission data obtained by exposure of the array to PNA solutions of varying concentration, and protein fractions of nut butters (Table S10), were assigned using the above model (Table S4). The unknown analytes were assigned with 100% accuracy (Table S11). Solutions of PNA with concentrations of 62.5–7.8 μM were assigned correctly. The protein fractions of peanut butter and mixed nut butter were assigned as PNA.

### Discrimination of Bacteria Using Emission Change Ratio

Luria–Bertani medium (10 g L^–1^ tryptone, 10 g L^–1^ NaCl, 5 g L^–1^ yeast; 5 mL aliquots) was inoculated from glycerol stocks of each bacterial strain. VRE culture was supplemented with vancomycin (4 μg mL^–1^). *E. coli* MG1655 culture was supplemented with kanamycin (30 μg mL^–1^). Cultures were grown with shaking at 180 rpm at 37 °C for 19 h. Cells were isolated by centrifugation (2500 *g*, 10 min) and resuspended in PBS (137 mM NaCl, 2.7 mM KCl, 10 mM Na_2_HPO_4_, 1.8 mM KH_2_PO_4_), pH 7.4 (5 mL).

Solutions of receptors **P3-(carbohydrate)** were prepared at 5.0 μM concentrations in PBS pH 7.4. Solutions were transferred to a 96-well plate (100 μL/well, 5 replicates per receptor) and emission spectra were recorded (λ_ex_ 470 nm, em 500–700 nm). A 10 μL aliquot of bacterial suspension was added to each well. The plate was shaken (3 × 10 s) and left at 21 °C for 15 min before emission spectra were acquired as before. The change in emission in response to bacterial addition was calculated:
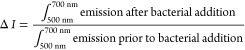


To assess the effects of dilution on emission of receptors, 10 μL aliquot of HEPES buffer was added to each of the receptors (5 replicates). Through visual inspection the effects of dilution were judged to be minimal and similar across all receptors. Linear discriminant analysis (LDA) was performed in SPSS (IBM). Raw data and LDA can be found in the Supporting Information, Section 4.2.

## Results and Discussion

**Scheme 2:**
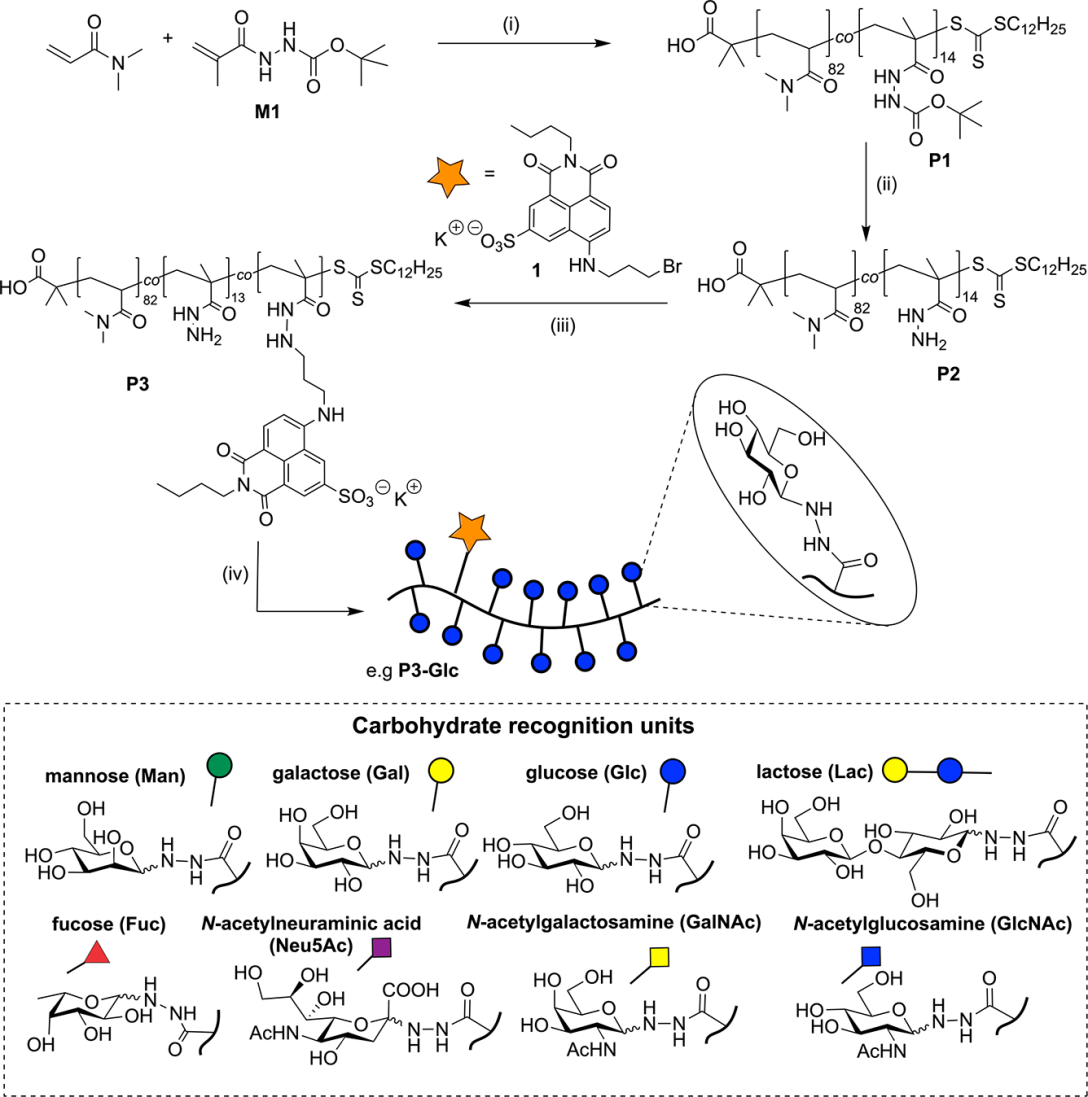
Preparation of Sensor Array (i) *S*-Dodecyl-*S*’-(α,α′-dimethyl-α″-acetic acid)trithiocarbonate, α,α′,-azoisobutyronitrile, DMF, 70 °C, 17 h; (ii) trifluoroacetic acid, CH_2_Cl_2_, 2 h; (iii) H_2_O, rt, 18 h; (iv) carbohydrate, 100 mM NH_4_OAc, pH 4.5, rt, 18 h.

Our receptors were constructed on the polymer backbone **P1** (Scheme 2), accessed via the RAFT copolymerization of *N*,*N*-dimethylacrylamide and **M1**, a methacrylamide derivative bearing BOC-protected acylhydrazide functionality. **P1** displayed an overall degree of polymerization of 97, with the two monomer units incorporated in a 5:1 ratio, respectively. Removal of the protecting groups yielded copolymers with pendant acylhydrazide functionalities, which were first used to install the naphthalimide derivative **1**, prepared in a 3-step synthetic route (Scheme 1). 4-Amino-1,8-naphthalimides are environmentally sensitive fluorophores which display high photostability and visible excitation/emission wavelengths.^37,38^ An average of one naphthalimide moiety was installed onto each polymer chain and the remaining pendant acylhydrazide units were used to install multiple copies of one of eight carbohydrates (Scheme 2),^39,40^ generating an array of eight fluorescent glycopolymers, which we expected to interact with a range of lectin substrates, including those displayed on bacterial cell surfaces. The spectra of fluorescent glycopolymers within the array show the characteristic green emission of naphthalimides (Figure 1(a), Figure S3).

To probe the underpinning recognition behavior of our sensor array, we initially focused on the discrimination of a selection of model lectins as a proof-of-concept study. A library of five lectins was selected for this study (Figure 1(b)), chosen to represent a range of carbohydrate binding preferences. Concanavalin A (Con A)^41^ is isolated from *Canavalia ensiformis* (Jack bean) and exists as a tetramer of four 26 kDa subunits which each display a binding site for mannose or glucose residues. The peanut agglutinin (PNA) and soybean agglutinin (SBA) are similarly sized tetrameric proteins which typically recognize galactosyl derivatives at one site per subunit.^42,43^ The wheat germ agglutinin (WGA) is generally described as recognizing *N*-acetyl glucose terminated sugars, but will also recognize other *N*-acetylated carbohydrates including *N*-acetyl galactose and *N*-acetylneuraminic acid at multiple recognition sites.^44^ The *E. coli* heat labile toxin (LTB)^45^ is a pentameric protein which displays carbohydrate recognition behavior similar to that of the cholera toxin, recognizing the carbohydrate portion of the GM1 ganglioside with high affinity at five sites across the surface of the pentamer, but also binding to its constituent fragments such as galactose and *N*-acetylneuraminic acid with much lower affinities. We proposed that these lectins would interact with our multivalent polymeric receptors to varying degrees, and that these recognition events would induce changes in the local environment of the naphthalimide fluorophore, generating a unique response pattern that could be attributed to each lectin.

Our experimental protocol for assessing the response of the array to lectin analytes is summarized below, with full experimental details provided in the Experimental Section, and data presented in the Supporting Information (Section 2). Briefly, we recorded emission spectra for each receptor in the array at 5.0 μM concentration (6 replicates), in buffer conditions suited to the lectin under study. Receptors were then exposed to analytes (125–250 μM subunit concentrations) in the same buffer and emission spectra were recorded again for each solution. As a control experiment, an equivalent volume of buffer was added to each receptor in the array (6 replicates), to exclude effects of dilution on emission behavior. In these cases, minimal changes in the emission of the glycopolymers were observed. Generally, we observed a decrease in the emission of the naphthalimide fluorophore with addition of analytes (Figure 1(c)), with the extent of quenching dependent on the combination of analyte and receptor (Table S2, Figure S4). We propose that interactions between our multivalent glycopolymers and lectins led to the formation of aggregates in solution in which emission behavior is altered.^46^ For the most part, the carbohydrate recognition sites on these lectins point outward, potentially promoting aggregation upon exposure to multivalent receptors. Broad, nonuniform particle size distributions were observed when selected lectin analytes and complementary glycopolymers were combined during dynamic light scattering analyses, suggesting aggregation (Figure S10).

The response of receptors within the array was assessed in terms of integrated emission after lectin exposure compared to its initial integrated emission (Table S2). This data set was subjected to linear discriminant analysis (LDA)^19,47^ to investigate discrimination between analytes. LDA is a multivariate statistical technique which analyses variance within the data provided (the “training set”), constructing a mathematical model which assigns the data into distinct groupings based on the combination of linear discriminant functions that describe each result. These linear discriminant functions, or factor scores, represent linear combinations of the responses of the receptors to each lectin, and the model constructed can be used for predictive purposes, i.e. to assign unknown analytes to one of these groupings. LDA enabled effective discrimination of the analytes, shown graphically (Figure 1(d)), with 94.1% of the variance in the data accounted for by the first linear discriminant function. This analysis enabled classification of the lectins with 100% accuracy. The predictive power of the model was confirmed by a “leave-one-out” validation procedure in which each result is excluded from the model, and the linear discriminant functions computed using the rest of the data set are used to assign its identity. Using this procedure, analytes were identified with a high degree of accuracy (96.7%), with a single discrepancy arising from a misclassification of one SBA replicate to Con A.

To further explore the limits of our array and probe the mechanism behind discrimination, we included BSA as a nonspecific binding analyte in the array. We found vastly different behavior of the array in response to this protein, showing 2–4 fold increases in fluorescence intensity, rather than aggregative quenching (Table S3), reflecting the environmental polarity change of the naphthalimide fluorophore. The array was able to accurately classify and discriminate BSA 100% of the time, and showed large separation of this analyte in the discriminant functions (Figure S5). To assess whether the discriminatory performance of the array could be replicated without the requirement to assess the emission of receptors prior to analyte exposure, we analyzed the raw integrated emission intensity after analyte addition (I), rather than the change in integrated emission intensity (I/I_0_) (Supporting Information Section 2.2). The response of the array again enabled the discrimination of all five lectins (Figure S7). While a more complete understanding of the mechanism of discrimination can be gained by analyzing the emission of receptors before and after analyte exposure, this simplified approach demonstrates the utility of the array as a method to enable convenient, rapid identification of lectins. The detection of proteins associated with foodstuffs often implicated in allergic reactions (peanuts, soy, wheat) could present opportunities for the development of devices to detect such allergens in processed foods.

**Figure 2:**
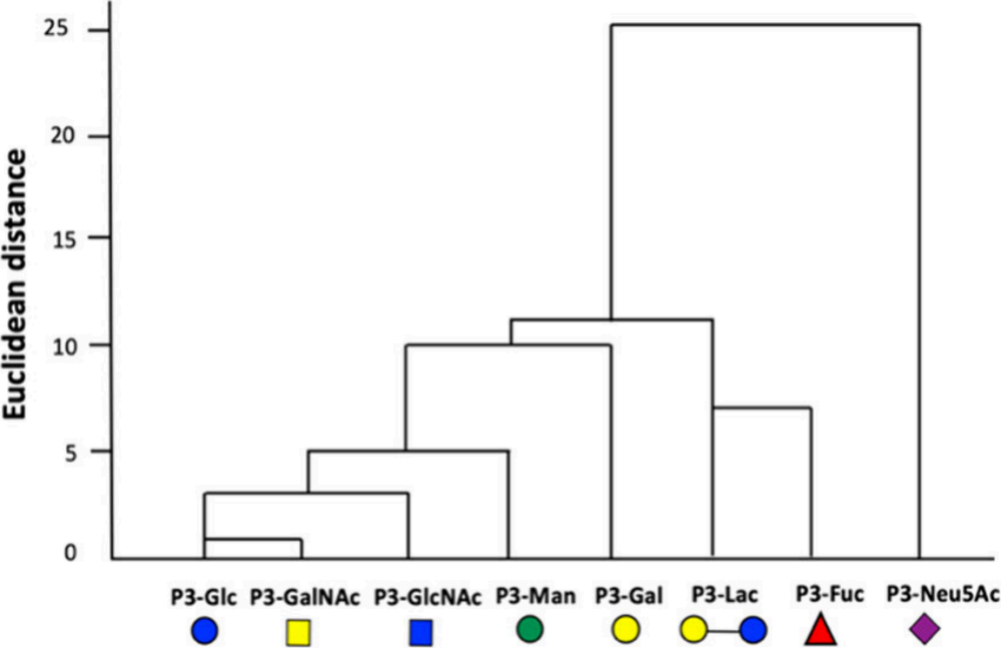
Dendrogram produced through hierarchical cluster analysis (HCA) of receptor responses to lectin addition.

We next explored the underlying mechanism of discrimination using hierarchical cluster analysis (HCA),^48^ a statistical technique that can identify groups of similar analytes or sensors in a stepwise clustering process.^49^ HCA of the receptor responses to analyte addition suggested that the discriminatory power of this sensor array is derived from structural differences between the carbohydrate recognition elements (Figure 2, Figure S8). The response of the sialic acid functionalized glycopolymer **P3-Neu5Ac** is immediately distinguished from that of all other receptors. This carbohydrate residue is distinct from the others in bearing a carboxylic acid functionality and a nine- carbon backbone (Scheme 2). Subsequent clustering distinguishes the behavior of the disaccharide receptor **P3-Lac** and deoxyhexose **P3-Fuc** from the hexose based receptors (**P3-Glc**, **P3-Gal**, **P3-Man**, **P3-GlcNAc**, **P3-GalNAc**), with this set displaying less diversity in their responses. This analysis suggests that the sensor array could be streamlined to incorporate fewer recognition motifs, for example by reducing the number of hexoses employed, presenting a route to simplified diagnostic tools. Indeed, performing LDA analysis on a subset of the data set, incorporating responses from 4 sensors (**P3-Neu5Ac**, **P3-Fuc, P3-Lac**, **P3-Gal**), produced the same level of analyte discrimination as achieved using eight sensors. Using these four sensors, the lectin analytes were classified with 100% accuracy, with 96.7% of cross-validated analytes identified correctly (Figure S9). This analysis further demonstrates the potential of array-based approaches to inform the design of specific receptors for lectins, by highlighting structural elements which will improve the selectivity of complexation for a particular lectin.

**Figure 3:**
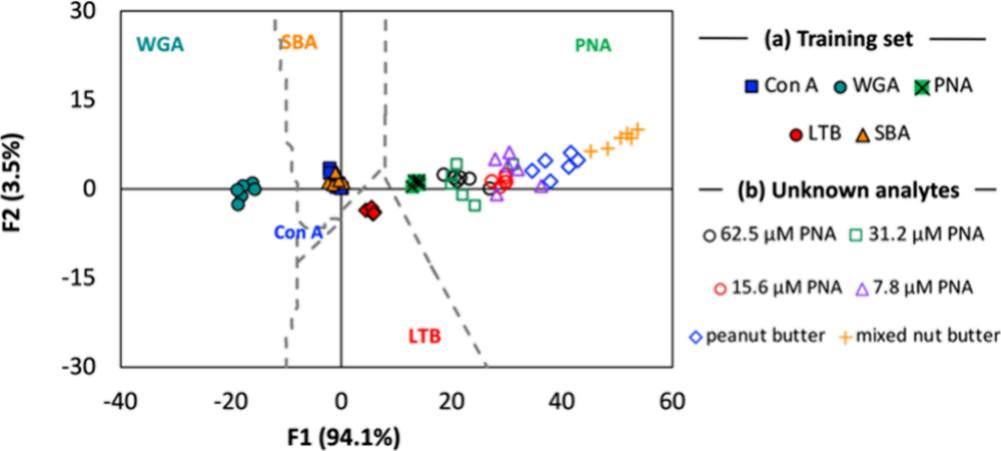
Canonical LDA score plot with overlaid territorial map (gray dashed lines) for the analysis of (a) the original training set, and (b) unknown analytes overlaid with the training set. The third score function (F3) has been approximated to zero to allow visualization in two dimensions.

Having established that our sensor array could discriminate lectin analytes, we next wished to assess the effect of varying analyte concentration on identification–an important consideration if the array is to be used to identify analytes in unknown samples. Using the same experimental procedure as previously employed, receptors within the array (5.0 μM) were exposed to a range of PNA concentrations from 7.8 μM to 62.5 μM in a 2-fold dilution series. The factor scores for these analytes were calculated using the model constructed earlier with purified lectin analytes (Table S4). In each case, the analytes were identified as PNA, with score functions located within the boundaries defined by LDA analysis of the training set (Figure 3).

Encouraged by the ability of the sensor array to correctly identify analytes across a range of concentrations, we next explored its ability to identify a lectin in a more complex environment than that presented by a solution of a single protein. Biological samples such as clinical isolates contain complex mixtures of proteins and other biomolecules, which could frustrate the ability of the sensor array to identify the relevant lectin. To model a complex environment in which lectin identification would be advantageous, we initially selected peanut butter, a complex mixture of proteins, fats and salts. The protein fractions of peanut butter, and a mixed nut butter containing peanuts, almonds and cashew nuts, were extracted into aqueous buffer. The effect of these solutions on the emission of the receptors within the array was assessed as before, and factor scores were calculated. The analytes were all correctly identified as PNA using the complete sensor array. Additionally, analysis using raw emission data after analyte addition (I) rather than change in emission intensity I/I_0_ (Table S10), and analysis using only the four receptors identified by HCA as driving discrimination (**P3-Neu5Ac**, **P3-Fuc**, **P3-Lac**, **P3-Gal**) (Figure 2, Table S11), also successfully identified solutions of varying PNA concentration and nut butter protein fractions with 100% accuracy.

The demonstrated ability of the glycopolymer sensor array to identify model lectin analytes at different concentration ranges and in complex mixtures suggested that it could discriminate bacteria based on differences in surface lectin composition. A diverse range of bacteria produce surface lectins, often called adhesins, that can interact with carbohydrate motifs displayed on epithelial surfaces, and within exopolysaccharide matrices produced in established infections.^4,50,51^ With this application in mind, we explored the response of the array to a selection of human pathogenic bacteria of different genera with significant differences in their surface properties.

*Salmonella enterica* is a Gram-negative bacterium which is a common cause of gastrointestinal disease. The *S. enterica* serovar Typhimarium used here is the causative agent of typhoid fever, and displays virulence traits including the production of adhesins and biofilm related proteins.^52^*Escherichia coli* K-12 MG1655 is a laboratory strain which approximates wild-type *E. coli* strains associated with diarrheal disease.^53^ The fimbriae displayed on this strain of *E. coli* are capped with the FimH lectin that binds to terminal mannose units on epithelial cell glycoproteins to initiate infection.^54^*Enteroccocus faecium* is a Gram-positive bacterium commonly found in the mammalian gastrointestinal tract, but some strains have emerged as nosocomial pathogens of concerning prevalence.^55^ Of particular concern is the emergence of resistance to antibiotics including vancomycin, typically viewed as an antibiotic of last resort.^56^ In this study both vancomycin sensitive *E. faecium* (VSE) and vancomycin resistant *E. faecium* (VRE) strains were investigated. *Pseudomonas aeruginosa* is a Gram-negative bacterium typically found in soil, which can cause opportunistic infections in immunocompromised individuals as discussed above.^57^*P. aeruginosa* surface proteins PA-IL (LecA) and PA-IIL (LecB) bind galactose-terminated and fucose-terminated glycans, respectively.^50^ The PAO1 strain used here was initially isolated from a wound infection, and is known to cause respiratory infection and is commonly associated with cystic fibrosis and ventilator associated pneumonia,^58,59^ while PA14 is a more virulent strain, with mutations in genes associated with adhesion and motility, frequently implicated in wound infections.^60^

Bacteria were grown to saturation in nutrient-rich medium before the cells were isolated by centrifugation and resuspended in phosphate buffered saline (PBS) at pH 7.4. We recorded emission spectra for each glycopolymer within the array at 5.0 μM in PBS pH 7.4 (5 replicates), before and after the addition of bacteria, and the data was analyzed as described above (Supporting Information, Section 4.2, Table S13).

**Figure 4:**
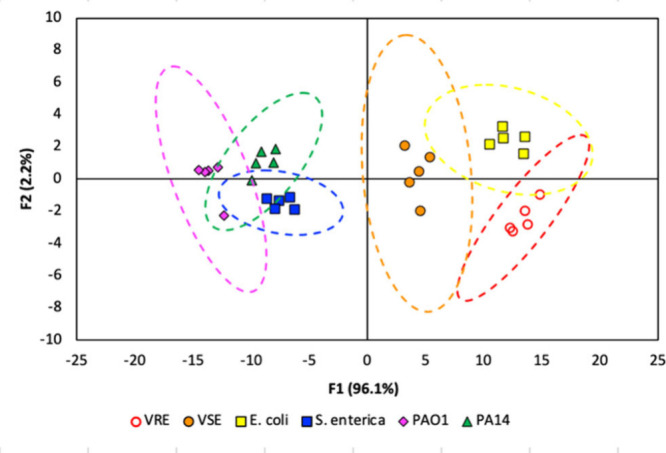
Canonical LDA score plots for analysis of a selection of bacteria performed in quintuplicate (5.0 μM receptors, pH 7.4). The pairing of the first (F1) and second (F2) factors is shown. Dashed lines indicate 95% confidence intervals.

The resulting LDA analysis showed 98.3% of the variance within the data set can be accounted for by the first and second linear discriminant functions, and bacteria could be classified with 100% accuracy. The “leave-one-out” validation procedure identified the bacteria with 90% accuracy, with misclassifications arising from one assignment each of gastrointestinal bacteria VRE and VSE to *E. coli* K-12 MG1655, and, interestingly, a misclassification between seemingly more different PA14 and *S. enterica* ser. Typhi. A large section of the PAPI-1 gene cluster, which is present in PA14 and thought to partially account for its higher virulence compared to PAO1, displays notable similarity to open reading frames present in *S. enterica* ser. Typhi.^60^ Notably, the sensor arrays also discriminated between vancomycin-sensitive and vancomycin-resistant bacteria VSE and VRE. While these *Enterococci* are genetically distinct from one another, vancomycin resistance is conferred to Enterococci through changes in the bacterial envelope. Vancomycin binds to d-Ala-d-Ala motifs within peptidoglycan, which are replaced by d-Ala-d-Lac in resistant strains,^61^ leading to differing surface functionality. We note also that there was accurate discrimination between the two *Pseudomonas* strains (Figure 4; PAO1 (pink diamonds), PA14 (green triangles)), which are known to differ in their cell surface colonization behavior,^62^ supporting the hypothesis that differences in lectin recognition contribute to discrimination. These promising results suggest that this sensor array could be applied as a diagnostic tool for rapid discrimination of clinically relevant bacterial pathogens, including discrimination between antibiotic- susceptible and resistant strains.

With the aim of again reducing the number of glycopolymer sensors needed to achieve analyte discrimination, HCA was performed on the complete data set (Figure S11). Analysis indicated that discrimination was largely driven by **P3-Neu5Ac** and **P3-Fuc**, with the hexose-based glycopolymers contributing similar, moderate amounts of discrimination. A subset of four glycopolymer receptors was chosen, comprised of the polymers contributing most to discrimination (**P3-Neu5Ac**, **P3-Fuc**), along with disaccharide-based receptor **P3-Lac** and a representative hexose-based receptor, **P3-GalNAc**. While LDA of the resultant data set demonstrated good discrimination (96.7% accuracy), only 73.3% of cases were identified correctly during “leave-one-out” validation, representing a decrease in performance compared with that of the complete eight sensor array (Figure S12), suggesting that in this case a higher number of sensors is needed to achieve accurate classification of analytes.

## Conclusions

In summary, we have demonstrated that lectins from plant and bacterial sources with similar carbohydrate binding preferences, along with a selection of pathogenic bacteria, can be discriminated using an array of eight fluorescent glycopolymers, which we generated using a conserved polymer scaffold and simple mono- or disaccharide sugars. The fluorescence response pattern produced by the array upon exposure to analyte has been analyzed by LDA, enabling the discrimination of lectins and their identification at varying concentrations, and within complex mixtures. The analysis can be further refined to identify analytes using just four receptors in some cases. We have demonstrated the ability of our sensor array to discriminate pathogenic bacteria of clinical importance and notable concern, and believe that this straightforward approach could enable the rapid identification of pathogens and their virulence profile, an application we will explore in our continuing investigations.

## Supporting Information Available

Characterization; raw data; statistical analysis PDF)
