# Supplementary material for: A Glycopolymer Sensor Array That Differentiates Lectins and Bacteria
Source: Biomacromolecules. 2024 Oct 18;25(11):7466–74. doi: 10.1021/acs.biomac.4c01129 (PMC11558668; doi:10.1021/acs.biomac.4c01129)
Supplement: Supplementary file 1 — bm4c01129_si_001.pdf [file bm4c01129_si_001.pdf]

Supporting information

A glycopolymer sensor array that differentiates lectins and bacteria

Kathryn G. Leslie,[a,b] Katrina A. Jolliffe,[b,c,e] Markus Müllner,[b,d,e] Elizabeth J. New,[b,c,e] W. Bruce Turnbull,[f] Martin A. Fascione,[g] Ville-Petri Friman[h,i] and Clare S. Mahon* [a,b]

[a] *Department of Chemistry, Durham University, Durham, DH1 3LE*

[b] *School of Chemistry, University of Sydney, Sydney, Australia, NSW 2006*

[c] Australian Research Council Centre of Excellence for Innovations in Peptide and Protein Science, *University of Sydney, Sydney, Australia, NSW 2006*

[d] *Key Centre for Polymers and Colloids, School of Chemistry, University of Sydney, Sydney, Australia, NSW 2006*

[e] Sydney Nano Institute, *University of Sydney, Sydney, Australia, NSW 2006*

[f] *School of Chemistry and Astbury Centre for Structural Molecular Biology, University of Leeds, Leeds, LS2 9JT, UK*

[g] *Department of Chemistry and York Structural Biology Laboratory, University of York, York YO10 5DD, UK*

[h] *Department of Biology, University of York, York YO10 5DD, UK*

[i] *Department of Microbiology, Faculty of Agriculture and Forestry, Viikki Biocenter (POB 56), FI-00014 University of Helsinki, Finland*

Table of Contents

1. Synthesis and Characterisation ... 3
1.1 Synthesis of M1 ... 3
1.1 Characterisation of P1 ... 3
1.2 UV-Vis spectra of extracted protein fractions ... 4
1.3 Excitation and emission spectra for glycopolymers ... 5
2. Discrimination of model lectin library ... 6
2.1 Discrimination using emission change ratio ... 6
2.1.1 Data ... 6
2.1.3 LDA ... 8
2.2 Discrimination using raw emission data ... 8
2.2.1 Data ... 9
2.2.2 LDA ... 10
2.3 Reducing the array to 4 sensors ... 11
2.4 Aggregate Characterisation by DLS ... 13
3. Discrimination within varied and complex samples ... 14
3.1 Discrimination using emission change ratio ... 14
3.1.1 Data ... 14
3.2 Discrimination using raw emission data ... 16
3.2.1 Data ... 16
3.2.3 LDA results ... 17
3.3 Using the reduced 4 sensor array ... 17
4. Discrimination of bacteria ... 18
4.1 Bacterial strain details ... 18
4.2 Discrimination using emission change ratio ... 18
4.2.1 Data ... 18
4.2.3 LDA ... 19
4.3 Reducing the array to 4 sensors ... 20
4.3.1 HCA analysis ... 20
4.3.2 Data ... 20
4.3.3 LDA ... 21
5 NMR spectra ... 23
6 References ... 36
7 Appendix ... 36

1. Synthesis and Characterisation

1.1 Synthesis of M1

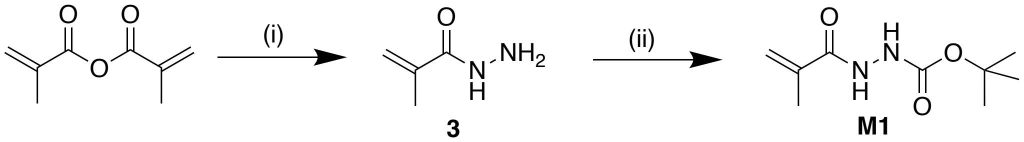

3 M1

Scheme 1 Preparation of **M1**. (i) $N_2H_4.H_2O$, $CHCl_3$, rt, 1 h. (ii) Di-*tert*-butyl dicarbonate, THF, rt, 24 h.

Methacryloyl hydrazide[1] (**3**)

Synthesis was adapted from a published procedure.[2] Methacrylic anhydride (9.6 mL, 65 mmol) in $CHCl_3$ (50 mL) was added dropwise to hydrazine hydrate (13.2 mL, 27 mmol) at 0 °C. The mixture was left to stir at room temperature for 1 h, then the organic fraction was removed. The aqueous fraction was extracted with $CHCl_3$ (3 × 25 mL) and the combined organic extracts were dried over $MgSO_4$ and evaporated to dryness, yielding a while solid. The product was recrystallized from 10:1 toluene : CH_2Cl_2 and isolated as colorless, needle-like crystals (2.65 g, 41%). ^{1}H NMR (300 MHz, $CDCl_3$): δ 1.95 (s, 3H, CH_3), 3.98 (s, 2H, NH_2), 5.35 (s, 1H, CH_2), 5.71 (s, 1H, CH_2), 7.39 (s, 1H, NH). ^{13}C NMR (75 MHz, $CDCl_3$/MeOH-d4): δ 169.4, 137.8, 120.5 18.0. Melting point 84 – 86 °C (84 – 86 °C[1])

***tert*-Butyl 2-methacryloylhydrazine-1-carboxylate** (**M1**)

Synthesis was adapted from a published procedure.[2] Di-*tert*-butyl dicarbonate (5.19 g, 24 mmol) in THF (50 mL) was added dropwise to **2** (2.31 g, 24 mmol) in THF (100 mL). The reaction mixture was left to stir at room temperature for 24 h before it was evaporated to dryness. The white solid obtained was dissolved in CH_2Cl_2 (100 mL) and washed with sat. $NaCl_{(aq)}$ (100 mL). The organic fraction was dried over $MgSO_4$ and evaporated to dryness. **M1** was recrystallised from 10:1 toluene : CH_2Cl_2 and isolated as white crystals (2.65 g, 55 %). ^{1}H NMR (300 MHz, $CDCl_3$): δ 1.47 (s, 9H, C(C*H*$_3$)$_3$), 1.98 (s, 3H, C*H*$_3$), 5.42 (s, 1H, C*H*$_2$), 5.81 (s, 1H, C*H*$_2$), 6.70 (s, 1H, NHN*H*CO), 7.89 (s, 1H, N*H*NHCO). ^{13}C NMR (75 MHz, $CDCl_3$): δ 167.5, 155.8, 137.7, 121.4, 81.8, 28.1, 18.4. Melting point: 116 - 118 °C

1.1 Characterisation of P1

Table S1 Characterisation of copolymer **P1**. ^{a} As determined by conversion analysis using ^{1}H NMR spectroscopy. m = number of DMA monomer units, determined by dimethyl signal at ~3.0 ppm; n = number of M1 monomer units, determined by Boc and backbone methyl signal at ~1.0 ppm. ^{b} As determined by gel permeation chromatography in DMAc + 0.05% w/w BHT + 0.03 % w/w LiBr (1.0 mL/ min) calibrated against near monodisperse poly(methyl methacrylate) standards (PSS, Germany). AIBN: α,α'-azobis(isobutyronitrile), DMA: *N*,*N*-dimethylacrylamide DDMAT: *S*-1-dodecyl-*S'*-(α,α-dimethyl-α''-acetic acid)trithiocarbonate.

Polymer	**DDMAT** / eq.	**AIBN** / eq.	**DMA** / eq.	**M1** / eq.	Conversion^a	m^a	n^a	n:m^a	M_n^a / g mol^{-1}	M_w^a / g mol^{-1}	M_w^b / g mol^{-1}	PDI (M_w/ M_n)
**P1**	1	0.2	85	15	97 %	82	14	5:1	11,500	13,500	14,700	1.09

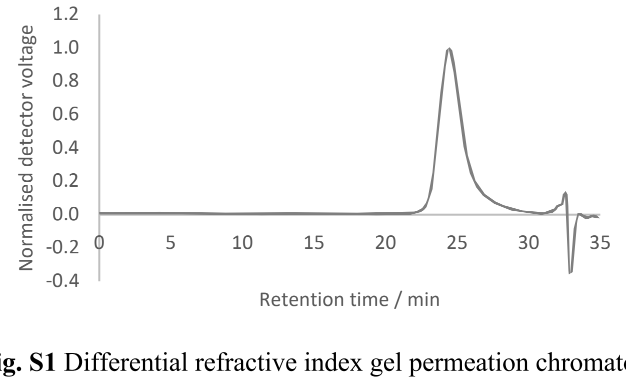

Fig. S1 Differential refractive index gel permeation chromatography (GPC) trace of P1 in dimethylacetamide (1.0 mL/min, containing 0.05% w/w BHT and 0.03 % w/w LiBr).

1.2 UV-Vis spectra of extracted protein fractions

Isolation of protein fractions of nut butters

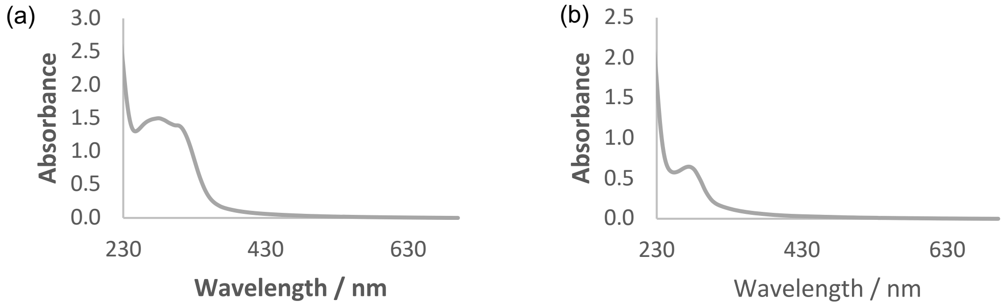

Fig. S2 UV-Vis spectra of protein fractions extracted from (a) peanut butter; (b) mixed nut butter.

1.3 Excitation and emission spectra for glycopolymers

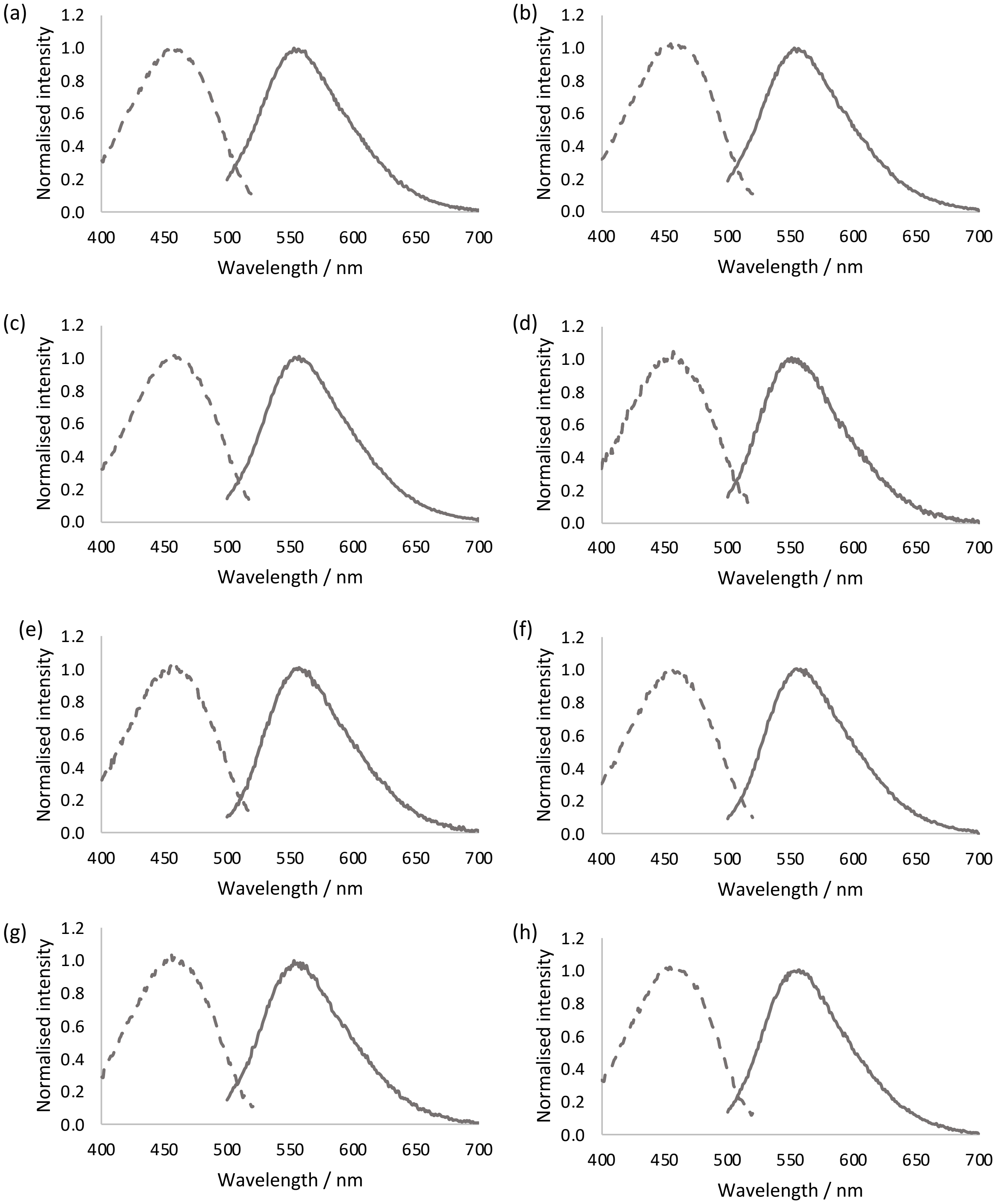

Fig. S3 Excitation (dashed line) and emission (solid line; λ_{ex} 555 nm) spectra of (a) **P3-Gal**; (b) **P3-Lac**; (c) **P3-Fuc**; (d) **P3-Neu5Ac**; (e) **P3-Man**; (f) **P3-Glc**; (g) **P3-GalNAc**; (h) **P3-GlcNAc**.

2. Discrimination of model lectin library

The lectins selected have the following overlapping carbohydrate recognition preferences:

Con A binds to mannosides and glucosides, e.g. α-Me-mannose (K_d 130 μM), α-Me-glucose (K_d 423 μM).[3]
PNA recognises a range of mono- , di- and oligo- saccharides including MeαGal (K_d 556 ± 28 μM), MeβGal (K_d 1 ± 0.05 mM), lactose K_d 709 ± 35 μM).[4]
LTB displays very similar carbohydrate recognition preferences (at the primary site) to the related cholera toxin (CTB),[5-6] which in addition to its natural ligand GM1 has been demonstrated to recognise**:** MeβGal (K_d 14.8 ± 1.6 mM), MeβGalNAc (K_d 118 ± 12 mM), *N*-acetylneuraminic acid (K_d 210 ± 100 mM).[7]
WGA recognises *N*-acetyl glucosamine (GlcNAc) terminated disaccharides (K_d 165 μM),[8] GalNAc (minimal agglutination concentration 200 mM),[9] Neu5Ac (minimal agglutination concentration 60 mM),[10] GlcNAc (minimal agglutination concentration 10 mM).[9]
SBA has been demonstrated to bind to carbohydrates including MeβGal (K_d 2 ± 0.2 mM), MeβGalNAc (K_d 45 ± 10 μM) and lactose (K_d 5 ± 1 mM).[11]

2.1 Discrimination using emission change ratio

2.1.1 Data

Table S2 Emission ratio change data used for LDA and construction of training set.

Lectin	P3-Gal	P3-Lac	P3-Fuc	P3-Neu5Ac	P3-Man	P3-Glc	P3-GalNAc	P4-GlcNAc
**Con A**	0.6600164	0.7907184	0.7331636	0.6779318	0.7178214	0.7306045	0.7721068	0.7227628
**Con A**	0.7318029	0.7744803	0.7834078	0.6547265	0.7056415	0.7444893	0.8133333	0.6975563
**Con A**	0.7534700	0.7999594	0.7928446	0.6299403	0.6867309	0.7572591	0.7951182	0.7255001
**Con A**	0.7471492	0.7874354	0.8348354	0.6273160	0.7343496	0.7354543	0.7492416	0.6796625
**Con A**	0.7503106	0.7887576	0.7730116	0.6452636	0.7778313	0.7560866	0.7786021	0.7059713
**Con A**	0.7004363	0.7827063	0.8409255	0.6709243	0.7096431	0.7087208	0.7394929	0.6480276
**WGA**	0.5658999	0.6351065	0.6431431	0.4887468	0.6756840	0.5688120	0.6066179	0.5350596
**WGA**	0.6001776	0.6478805	0.6538201	0.4871547	0.5463990	0.5564521	0.5445270	0.5222067
**WGA**	0.5634650	0.5970090	0.6292396	0.3950554	0.5292498	0.5435281	0.5782086	0.5739586
**WGA**	0.6121965	0.6163128	0.5996734	0.4081042	0.5396974	0.5270596	0.5590027	0.5215627
**WGA**	0.5772297	0.6671990	0.5787511	0.4949310	0.5121579	0.5323700	0.5797815	0.5295813
**WGA**	0.5247492	0.5420897	0.5642072	0.4382599	0.5014080	0.5202707	0.5223767	0.5457807
**PNA**	0.8588598	0.7847400	0.8502745	0.9015645	0.7688332	0.8064890	0.9049284	0.8838813
**PNA**	0.8383450	0.8219891	0.8564765	0.8589723	0.8503465	0.8457135	0.8860421	0.9119305
**PNA**	0.8360781	0.8009821	0.8757535	0.8626455	0.8199158	0.8438307	0.8239611	0.8736485
**PNA**	0.8491950	0.8405863	0.8752319	0.8310142	0.8109007	0.8835881	0.8954486	0.8256717
**PNA**	0.8507107	0.8254614	0.8820302	0.8132984	0.9214217	0.8557127	0.8314284	0.8597009
**PNA**	0.8345887	0.7909617	0.8789463	0.8815721	0.8356354	0.8563093	0.8825952	0.9227347
**LTB**	0.8736124	0.7701149	0.7935369	0.6813201	0.7211651	0.7704506	0.7832914	0.7892153
**LTB**	0.8719632	0.7825306	0.7673500	0.6842516	0.7216720	0.7692943	0.7827823	0.7812326
**LTB**	0.8852964	0.7857561	0.7633031	0.6862844	0.7537887	0.7748834	0.8087620	0.7837733
**LTB**	0.8640969	0.7849669	0.8270263	0.6627921	0.7226542	0.7449578	0.7727201	0.7775177

**LTB**	0.8658880	0.7595027	0.8199856	0.6997326	0.7622135	0.7651279	0.7801995	0.7334490
**LTB**	0.8862939	0.7864643	0.8016997	0.6701265	0.7276881	0.7853123	0.7763241	0.7619048
**SBA**	0.7061425	0.7122609	0.7405174	0.6817740	0.6662535	0.6840725	0.6903733	0.6477940
**SBA**	0.6887219	0.7154818	0.9182354	0.6691381	0.6874394	0.6933294	0.7416857	0.6660893
**SBA**	0.7179035	0.7031810	0.7314212	0.7139212	0.6868208	0.6636894	0.6899444	0.6799270
**SBA**	0.7251902	0.7307580	0.7564395	0.7106639	0.6678620	0.7056765	0.7025823	0.7133882
**SBA**	0.7122631	0.7404941	0.6983856	0.7322785	0.6883404	0.6897181	0.6931507	0.7211152
**SBA**	0.6392148	0.6878571	0.6548105	0.7046247	0.6977030	0.7244905	0.6938168	0.7344290
**buffer control**	1.0058227	1.0012013	0.9664917	0.9472614	0.9789263	0.9738333	0.9469821	0.9948308
**buffer control**	0.9853694	0.9899236	0.9802517	0.9921979	0.9634537	0.9654887	0.9597954	0.9490476
**buffer control**	0.9826865	0.9769143	0.9553617	1.0064906	0.9061173	0.9030168	0.9444316	1.0005680
**buffer control**	0.9690273	0.9687219	0.9383862	1.1108019	0.9630689	0.9470326	1.0028427	0.9731870
**buffer control**	0.9690069	0.9816396	0.9972694	0.9512480	0.9461792	0.9517252	0.9518454	0.9422970
**buffer control**	0.9590237	0.9897209	0.9253483	0.9799237	0.9329510	0.9456482	0.9679507	0.9458815

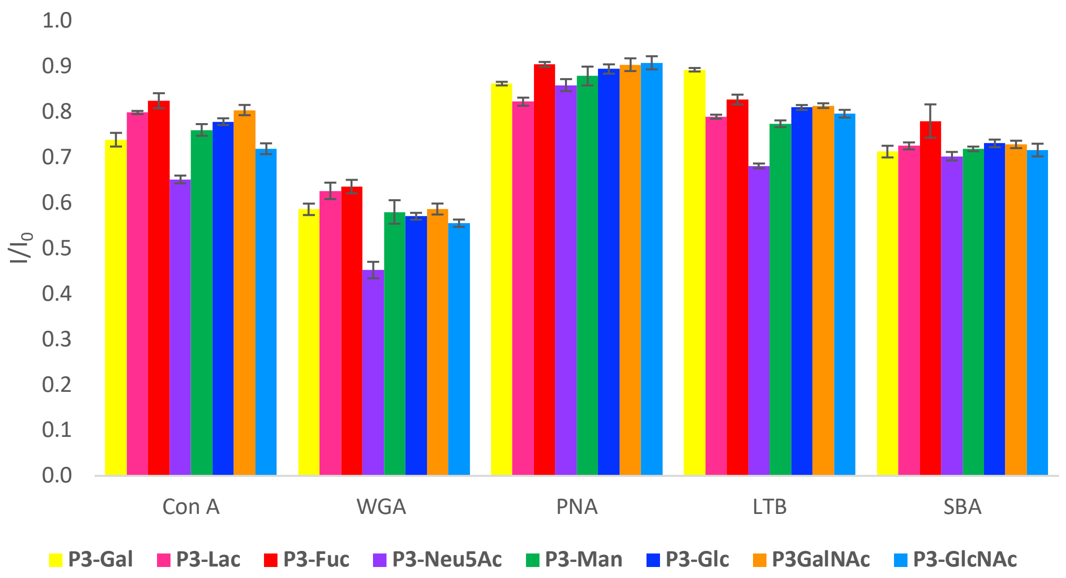

Fig. S4 Graphical illustration of data in Table S2 - change in glycopolymer emission upon addition of lectins. The error bars represent standard error.

Table S3 Emission ratio change data for BSA analyte with glycopolymer array.

Protein	P3-Gal	P3-Lac	P3-Fuc	P3-Neu5Ac	P3-Man	P3-Glc	P3-GalNAc	P4-GlcNAc
**BSA**	2.74178636	2.59477124	2.71321728	4.18093856	3.43858938	3.52327291	3.16758868	3.54131799
**BSA**	2.54586784	2.98304102	2.98430933	3.89721334	3.4480991	3.72306322	3.34053537	3.39848522
**BSA**	2.93414211	2.81771878	2.99216235	4.03052729	3.8427957	3.68148028	3.40142156	3.59824186
**BSA**	3.17625232	3.20562876	3.26544264	4.42356526	3.68848997	3.49832134	3.33945401	3.2292749
**BSA**	3.14406487	2.85005513	3.12985238	4.04857929	3.67347765	3.50992248	3.37567347	3.27167113
**BSA**	2.85667107	2.96351872	3.25247525	4.3679159	3.69564332	3.49226442	3.52109879	3.36265503

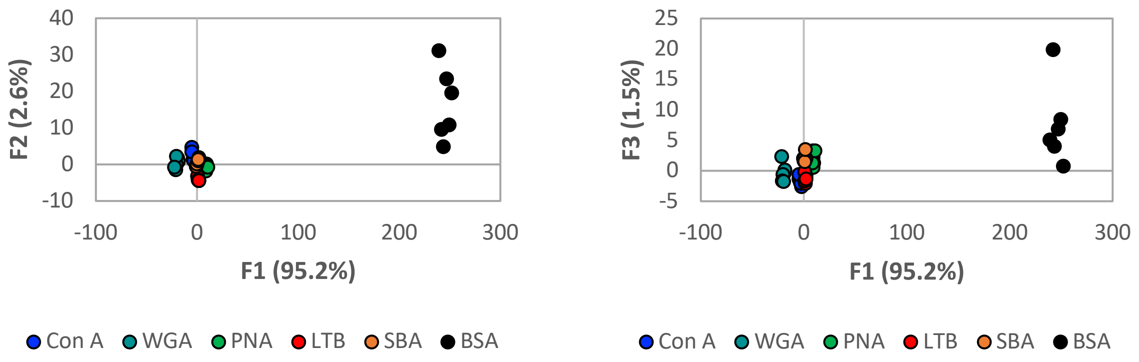

Fig. S5 Canonical LDA score plots for the analysis of lectins and BSA performed in sextuplicate (5.0 µM receptors, 125 – 250 µM lectin subunit, pH 7.4). The pairing of the first (F1) and second (F2) and first and third (F3) factors is shown in separate 2D plots.

2.1.3 LDA

Table S4 Canonical discriminant function coefficients for receptors obtained through LDA analysis of emission change ratio.

	Function			
	1	2	3	4
**P3-Gal**	21.395	-35.69	-12.686	-7.603
**P3-Lac**	-12.784	10.597	33.703	-10.005
**P3-Fuc**	11.227	6.961	-2.092	-0.142
**P3-Neu5Ac**	25.571	16.232	-20.773	-14.068
**P3-Man**	-1.404	-1.155	-0.471	13.351
**P3-Glc**	34.304	5.839	0.609	-21.61
**P3-GalNAc**	-4.366	1.851	20.982	15.499
**P3-GlcNAc**	17.905	-3.616	-1.972	23.445
**(Constant)**	-65.328	0.326	-14.288	0.455

Unstandardized coefficients

2.2 Discrimination using raw emission data

2.2.1 Data

Data below was used as a training set to construct a scoring model which enabled the identification of unknown samples.

Table S5 Raw emission data (counts per second) used for LDA and construction of training set

Lectin	P3-Gal	P3-Lac	P3-Fuc	P3-Neu5Ac	P3-Man	P3-Glc	P3-GalNAc	P4-GlcNAc
**Con A**	8817	8527	8944	10406	8543	10181	6509	8319
**Con A**	7048	8443	8965	10335	8059	11041	6361	7964
**Con A**	7111	8762	8541	11449	8321	11336	6903	8595
**Con A**	6920	9490	8541	10264	7892	10867	6555	8054
**Con A**	6719	9684	8363	11739	8012	11440	6615	8406
**Con A**	6588	9346	8303	11280	8264	10821	6694	8066
**WGA**	8291	9660	9599	11535	9478	11518	3670	4694
**WGA**	8789	10456	9415	11587	9653	12106	3527	5063
**WGA**	8430	10694	9062	11825	9740	12144	3100	4957
**WGA**	8774	10942	9347	11732	9548	12783	3384	4740
**WGA**	8472	10872	9192	11885	9157	12236	3515	4884
**WGA**	7845	10274	8597	11858	8985	12793	3244	4885
**PNA**	9285	8777	11660	11832	9329	11127	10128	9355
**PNA**	9254	10063	11184	11818	9020	11585	9799	9547
**PNA**	10296	9156	11631	11763	9858	11004	10066	9535
**PNA**	9758	9507	11561	12372	9829	11588	10299	9250
**PNA**	10110	10577	11304	11796	9307	10693	9727	9314
**PNA**	10192	9680	11581	12181	9791	11359	10112	10163
**LTB**	10624	11434	11524	11622	11615	12079	5223	5532
**LTB**	10624	11124	11324	10736	11455	11935	5266	5299
**LTB**	10350	10915	12070	11418	11619	12349	5434	5263
**LTB**	10491	11234	11853	11126	12479	12739	5474	5375
**LTB**	10453	11431	11729	11762	12563	12860	5495	4966
**LTB**	10398	11302	12957	11219	13018	12445	5617	5114
**SBA**	8622	12783	9196	14168	10191	14912	4212	6520
**SBA**	8983	12123	9400	12575	12780	13551	4332	5977
**SBA**	8958	11976	9616	12822	10787	13248	4477	6823
**SBA**	8864	12233	9969	12946	10631	13037	4485	6386
**SBA**	9206	12850	9562	12992	10642	13513	4628	6517
**SBA**	8499	12966	9511	13191	10420	13350	4647	5906
**buffer control**	7526	10777	9131	13584	8950	12128	7723	10585
**buffer control**	7799	10835	9292	12841	9768	12199	7307	9965
**buffer control**	8406	10665	8768	12123	9050	12203	7452	10570
**buffer control**	7822	11109	9883	12766	9305	13053	7721	10199
**buffer control**	8088	10636	9076	12302	9108	12018	7889	10141
**buffer control**	9082	11062	9919	12788	9796	12413	7865	10312

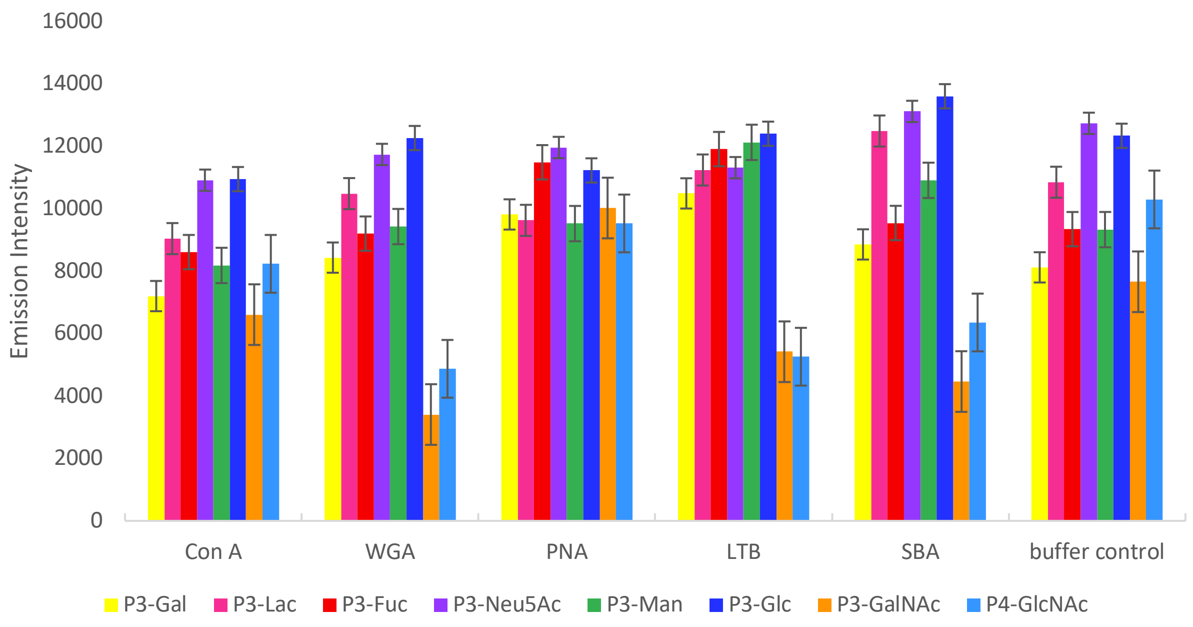

Fig. S6 Graphical illustration of data in Table S5 – raw emission data of glycopolymers after addition of lectins. Error bars represent standard error.

2.2.2 LDA

Table S6 Canonical discriminant function coefficients for receptors obtained through LDA analysis of raw emission data

	Function				
	**1**	**2**	**3**	**4**	**5**
**P3-Gal**	-0.001	0.001	0	0.001	0
**P3-Lac**	0	0	0.001	0	-0.001
**P3-Fuc**	-0.001	0.001	0.001	-0.001	0.002
**P3-Neu5Ac**	-0.001	0	0	0.002	0.001
**P3-Man**	0	0	0.001	-0.001	-0.001
**P3-Glc**	0	0	0	0	0
**P3-GalNAc**	0.005	0.002	-0.001	0.001	-0.002
**P3-GlcNAc**	0.003	-0.002	0.002	-0.001	0.002
**(Constant)**	-30.233	-28.984	-47.282	-6.744	-10.45

Unstandardized coefficients

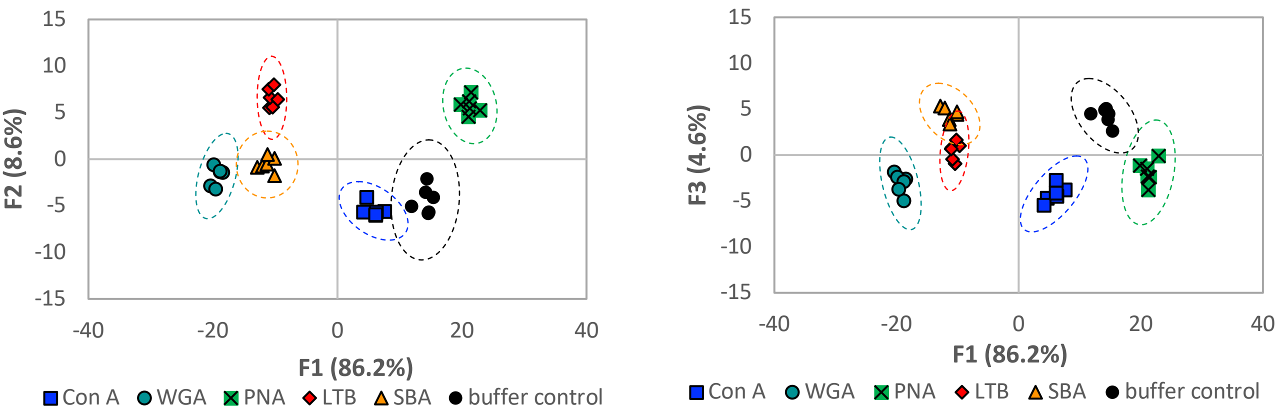

Fig. S7 Canonical LDA score plots for the analysis of lectins performed in sextuplicate (5.0 µM receptors, 125 – 250 µM lectin subunit, pH 7.4). The pairing of the first (F1) and second (F2) and first and third (F3) factors is shown in separate 2D plots.

2.3 Reducing the array to 4 sensors

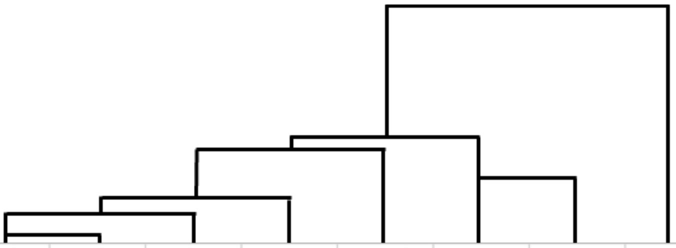

Repeat	Lectin	P3Glc	P3GalNAc	P3GlcNAc	P3Man	P3Gal	P3Lac	P3Fuc	P3Neu5Ac
19	LTB	0.7705	0.7833	0.7892	0.7212	0.8736	0.7701	0.7935	0.6813
20	LTB	0.7693	0.7828	0.7812	0.7217	0.872	0.7825	0.7673	0.6843
24	LTB	0.7853	0.7763	0.7619	0.7277	0.8863	0.7865	0.8017	0.6701
21	LTB	0.7749	0.8088	0.7838	0.7538	0.8853	0.7858	0.7633	0.6863
22	LTB	0.745	0.7727	0.7775	0.7227	0.8641	0.785	0.827	0.6628
23	LTB	0.7651	0.7802	0.7334	0.7622	0.8659	0.7595	0.82	0.6997
25	SBA	0.6841	0.6904	0.6478	0.6663	0.7061	0.7123	0.7405	0.6818
27	SBA	0.6637	0.6899	0.6799	0.6868	0.7179	0.7032	0.7314	0.7139
28	SBA	0.7057	0.7026	0.7134	0.6679	0.7252	0.7308	0.7564	0.7107
29	SBA	0.6897	0.6932	0.7211	0.6883	0.7123	0.7405	0.6984	0.7323
30	SBA	0.7245	0.6938	0.7344	0.6977	0.6392	0.6879	0.6548	0.7046
4	ConA	0.7355	0.7492	0.6797	0.7343	0.7471	0.7874	0.8348	0.6273
6	ConA	0.7087	0.7395	0.648	0.7096	0.7004	0.7827	0.8409	0.6709
2	ConA	0.7445	0.8133	0.6976	0.7056	0.7318	0.7745	0.7834	0.6547
3	ConA	0.7573	0.7951	0.7255	0.6867	0.7535	0.8	0.7928	0.6299
5	ConA	0.7561	0.7786	0.706	0.7778	0.7503	0.7888	0.773	0.6453
1	ConA	0.7306	0.7721	0.7228	0.7178	0.66	0.7907	0.7332	0.6779
26	SBA	0.6933	0.7417	0.6661	0.6874	0.6887	0.7155	0.9182	0.6691
14	PNA	0.8457	0.886	0.9119	0.8503	0.8383	0.822	0.8565	0.859
18	PNA	0.8563	0.8826	0.9227	0.8356	0.8346	0.791	0.8789	0.8816
15	PNA	0.8438	0.824	0.8736	0.8199	0.8361	0.801	0.8758	0.8626
13	PNA	0.8065	0.9049	0.8839	0.7688	0.8589	0.7847	0.8503	0.9016
16	PNA	0.8836	0.8954	0.8257	0.8109	0.8492	0.8406	0.8752	0.831
17	PNA	0.8557	0.8314	0.8597	0.9214	0.8507	0.8255	0.882	0.8133
9	WGA	0.5435	0.5782	0.574	0.5292	0.5635	0.597	0.6292	0.3951
10	WGA	0.5271	0.559	0.5216	0.5397	0.6122	0.6163	0.5997	0.4081
8	WGA	0.5565	0.5445	0.5222	0.5464	0.6002	0.6479	0.6538	0.4872
11	WGA	0.5324	0.5798	0.5296	0.5122	0.5772	0.6672	0.5788	0.4949
12	WGA	0.5203	0.5224	0.5458	0.5014	0.5247	0.5421	0.5642	0.4383
7	WGA	0.5688	0.6066	0.5351	0.6757	0.5659	0.6351	0.6431	0.4887

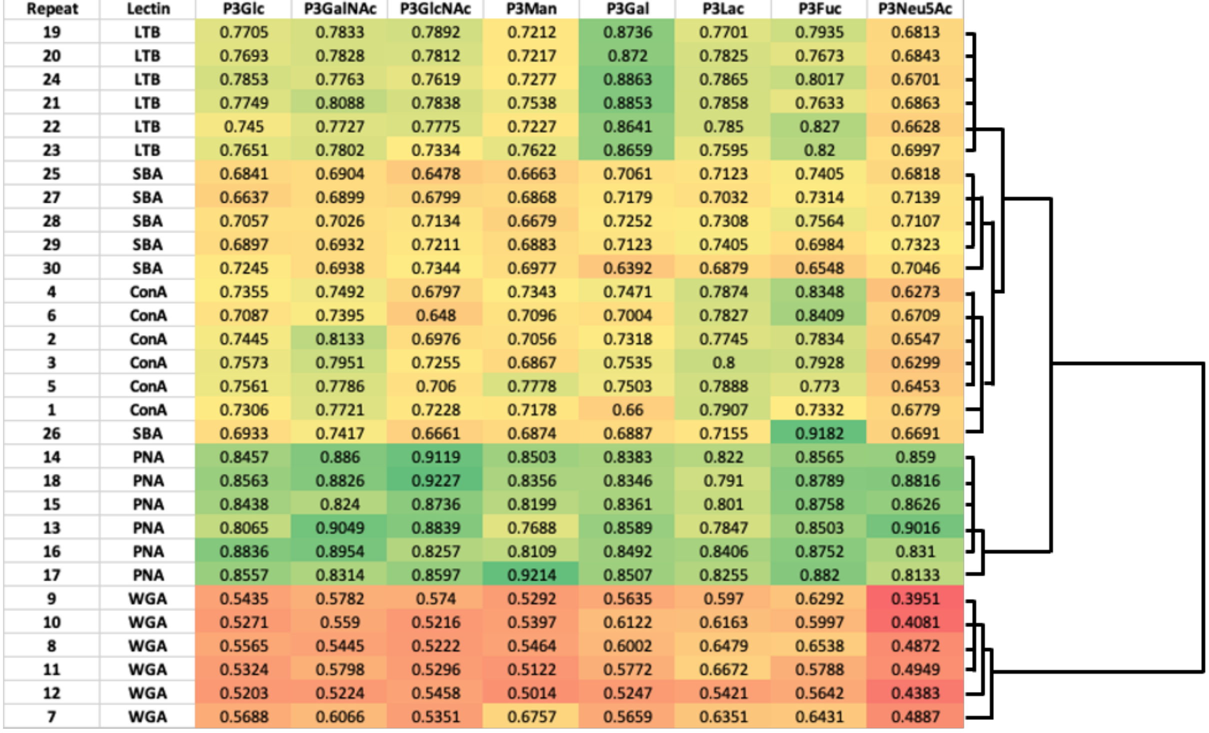

Fig. S8 Hierarchical cluster analysis (HCA) heatmap of glycopolymer responses to lectin analytes

Table S7 Data from 4 sensors used for LDA and construction of training set.

Lectin	P2NGal	P2NLac	P2NFuc	P2NSia
**Con A**	0.6600164	0.7907184	0.73316359	0.67793183
**Con A**	0.73180291	0.77448032	0.78340775	0.6547265
**Con A**	0.75347002	0.79995941	0.79284455	0.62994027
**Con A**	0.7471492	0.7874354	0.83483542	0.62731604
**Con A**	0.75031056	0.78875763	0.77301156	0.64526358
**Con A**	0.70043632	0.78270627	0.84092552	0.67092432
**WGA**	0.56589994	0.63510652	0.64314311	0.48874684
**WGA**	0.60017755	0.64788054	0.6538201	0.4871547
**WGA**	0.56346501	0.59700903	0.62923961	0.39505544
**WGA**	0.61219648	0.6163128	0.59967341	0.4081042
**WGA**	0.57722968	0.66719895	0.57875111	0.49493101
**WGA**	0.52474916	0.54208967	0.56420722	0.43825993
**PNA**	0.85885976	0.78474004	0.85027453	0.90156454
**PNA**	0.83834501	0.82198913	0.8564765	0.85897228
**PNA**	0.83607813	0.80098211	0.87575349	0.86264547
**PNA**	0.84919495	0.84058628	0.87523188	0.83101422
**PNA**	0.85071072	0.82546144	0.88203018	0.8132984
**PNA**	0.83458869	0.79096165	0.87894625	0.88157214
**LTB**	0.87361237	0.77011494	0.79353693	0.68132011
**LTB**	0.87196323	0.78253058	0.76734995	0.68425156
**LTB**	0.88529638	0.78575614	0.76330311	0.68628442
**LTB**	0.86409686	0.78496689	0.82702631	0.66279211
**LTB**	0.86588801	0.75950269	0.81998564	0.69973259
**LTB**	0.8862939	0.78646434	0.80169972	0.67012646
**SBA**	0.70614251	0.71226086	0.74051737	0.68177404
**SBA**	0.68872192	0.71548181	0.91823538	0.66913809
**SBA**	0.71790351	0.70318099	0.73142121	0.71392122
**SBA**	0.72519022	0.73075795	0.75643945	0.71066392
**SBA**	0.71226306	0.74049408	0.69838561	0.73227848
**SBA**	0.6392148	0.68785709	0.65481053	0.70462472

Table S8 Canonical discriminant function coefficients for receptors obtained through LDA analysis

	Function			
	**1**	**2**	**3**	**4**
**P3-Gal**	24.469	-34.974	-10.844	-0.216
**P3-Fuc**	7.145	7.814	1.76	19.111
**P3-Lac**	-3.875	11.628	43.961	-14.394
**P3-Neu5Ac**	29.816	16.795	-14.719	-5.137
**(Constant)**	-40.707	0.159	-16.053	-0.326

Unstandardized coefficients

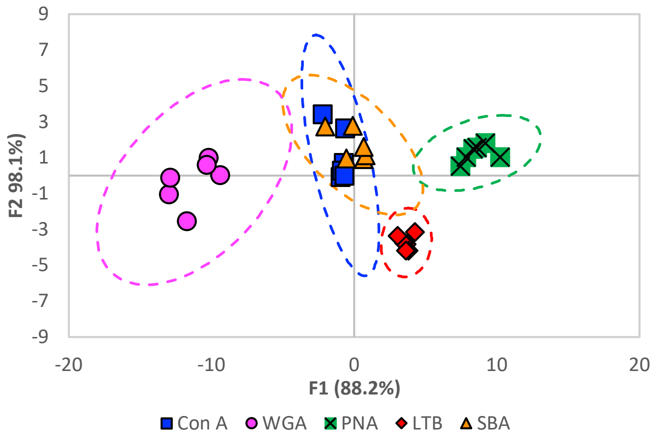

Fig. S9 Classification of analytes using LDA training set constructed using four glycopolymer sensors. Dashed lines denote 95% confidence intervals.

2.4 Aggregate Characterisation by DLS

Solutions of selected glycopolymers identical to those used for fluorescence analysis (500 µL @ 5.0 µM in 10 mM HEPES, 1 mM $MnCl_2$, 1 mM $CaCl_2$, pH 7.4), were subjected to DLS analysis. No significant scattering of light was observed in any case (count rates < 50 counts per second). Aliquots of lectins (50 µL @ 250 µM subunit concentration in the same buffer) were added to the glycopolymer solutions. After 15 min, the DLS analysis was repeated. Broad, non-uniform particle size distributions were observed in each case (Fig. S10), suggesting the presence of large aggregates in solution.

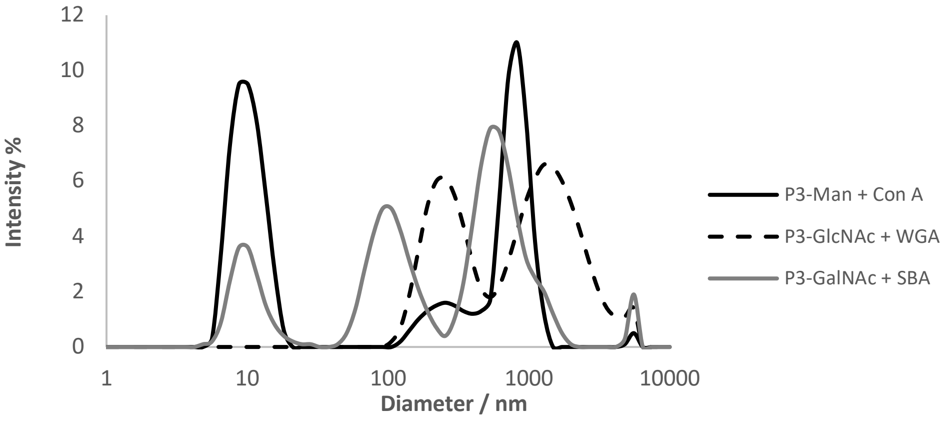

Fig. S10 Particle size distributions of mixtures of **P3-Man** and ConA, **P3-GlcNAc** and WGA, and **P3-GalNAc** and SBA

3. Discrimination within varied and complex samples

3.1 Discrimination using emission change ratio

3.1.1 Data

Table S9 Data used for identification of unknown analytes

Analyte	P3-Gal	P3-Lac	P3-Fuc	P3-Neu5Ac	P3-Man	P3-Glc	P3-GalNAc	P4-GlcNAc
**7.8 µM PNA**	1.092844905	0.91402107	1.08929039	1.148165643	0.933839567	1.05094512	0.998373869	0.998868778
**7.8 µM PNA**	0.89088474	0.889418099	1.021480835	1.119123761	1.01103826	1.02700556	1.022925764	1.041355119
**7.8 µM PNA**	0.970839201	0.946305357	1.050751204	1.113345225	1.053156434	1.003707136	1.012967581	1.00859375
**7.8 µM PNA**	0.956540228	0.974030287	1.041103052	1.115611676	1.066950149	1.00244721	1.024170683	0.925474832
**7.8 µM PNA**	0.997423096	0.960708834	1.071399512	1.087464477	1.059588012	1.064473971	1.049144362	1.001848592
**7.8 µM PNA**	1.02707302	0.959480621	1.048198347	0.940552017	1.084765957	1.014077533	1.062050026	1.080342352
**15.6 µM PNA**	1.0184658	0.991205756	0.950548328	1.098355569	1.031705378	1.020118428	1.033842376	1.01459854
**15.6 µM PNA**	1.015398778	0.974444606	1.049893624	1.01685761	1.028810324	1.032325115	1.014218364	1.028042678
**15.6 µM PNA**	0.988033139	0.94057725	0.99180275	1.023084171	0.997860963	0.986576699	1.007997092	1.011317495
**15.6 µM PNA**	1.001218663	0.950224452	1.075828123	1.077455717	0.993423113	0.985499163	1.023418149	1.018346112
**15.6 µM PNA**	0.976986177	0.858735263	0.988757278	1.035255406	0.979550276	1.029168481	0.945228805	1.001707712
**15.6 µM PNA**	1.016691395	0.893145027	1.004273777	1.04596835	1.012503168	0.986029205	1.039039907	0.998932479
**31.3 µM PNA**	0.87180287	0.829236111	0.952978454	0.851754769	0.958315279	0.942527923	0.850072385	0.97105093
**31.3 µM PNA**	0.913219165	0.742996096	0.925315036	0.875174662	0.939098559	0.956472663	0.929112999	0.949531624
**31.3 µM PNA**	0.8728821	0.897099139	0.971449247	0.886843358	1.073333884	0.944828559	0.931441465	0.99100295
**31.3 µM PNA**	0.856770105	0.882522223	0.945144172	0.979353979	1.017277487	0.937041737	0.996341246	0.958132956
**31.3 µM PNA**	0.913931495	0.871698371	1.012227539	1.08212723	0.970580667	1.017952664	0.948365527	1.091423532
**31.3 µM PNA**	1.016751583	0.906744829	0.94084726	0.868010403	0.928894472	1.004454023	0.937110378	0.979127421
**62.5 µM PNA**	0.915026146	0.890704598	0.952431067	0.900126024	0.941222914	0.975872072	0.949783868	0.918287327
**62.5 µM PNA**	0.94553411	0.807084823	0.986499131	0.916015014	0.926264938	1.041302912	0.941576962	0.976924817
**62.5 µM PNA**	0.866908926	0.845328947	0.915492958	0.847745522	0.935801354	1.046436518	1.02449434	0.954714776
**62.5 µM PNA**	0.869053264	0.82267874	0.963096371	0.914773669	0.991361072	0.911422077	0.931590866	0.988163951
**62.5 µM PNA**	0.922567645	0.812666445	0.923693271	1.00529661	0.853641698	0.947561837	0.892519504	0.877662679
**62.5 µM PNA**	0.852539376	0.871681104	0.971315284	0.859389454	0.943737096	0.938800057	0.874168708	0.940121493
**Peanut butter protein fraction**	1.051363827	0.998226351	0.954765379	1.339288937	1.080891089	1.028281143	1.080590717	1.036566289
**Peanut butter protein fraction**	1.109369133	1.141491072	1.081031787	1.216633267	1.110107571	1.179594862	1.182113369	1.141127781
**Peanut butter protein fraction**	1.097563129	1.045863382	0.964526762	1.411578947	1.184497705	1.111292162	0.910148976	1.044189682

**Peanut butter protein fraction**	1.042722147	1.078742001	1.038726196	1.110711431	1.067548884	1.107184628	1.093506494	1.095017023
**Peanut butter protein fraction**	1.145121951	1.078266442	1.072934199	1.231542949	1.039862205	1.036783854	1.150300778	1.109026369
**Peanut butter protein fraction**	1.084456349	1.073210459	1.072146884	1.394875966	1.070492765	1.061296252	1.063213345	1.087993335
**Mixed nut butter protein fraction**	1.151564232	1.20753507	1.282242541	1.511043819	1.299289261	1.153840356	1.17425897	1.177230711
**Mixed nut butter protein fraction**	1.150264801	1.273318872	1.286547016	1.506393862	1.32387495	1.176415094	1.383709687	1.306230201
**Mixed nut butter protein fraction**	1.187152985	1.192830939	1.283131607	1.629076748	1.215215399	1.070970177	1.154185729	1.209904333
**Mixed nut butter protein fraction**	1.135477661	1.162971606	1.189764973	1.640992379	1.23787865	1.14303444	1.158152936	1.221160216
**Mixed nut butter protein fraction**	1.144811504	1.214210998	1.189288066	1.393379907	1.164477336	1.12084843	1.162662623	1.163486966
**Mixed nut butter protein fraction**	1.103207698	1.050442542	1.071042042	1.500710227	1.147387895	1.115505148	1.076876422	1.177358491

3.2 Discrimination using raw emission data

3.2.1 Data

Table S10 Raw data used for identification of unknown analyte.

Lectin	P3-Gal	P3-Lac	P3-Fuc	P3-Neu5Ac	P3-Man	P3-Glc	P3-GalNAc	P4-GlcNAc
**7.8 µM PNA**	13395	12927	16640	7292	11560	14956	14121	11479
**7.8 µM PNA**	11308	12885	15217	6999	12182	14223	14055	11281
**7.8 µM PNA**	11719	15086	14617	6866	12145	14079	14217	11619
**7.8 µM PNA**	11247	14665	15932	6803	11793	14337	14449	10866
**7.8 µM PNA**	12386	14475	15801	6888	11985	14661	14346	11381
**7.8 µM PNA**	13278	14705	15854	6202	12746	14335	14309	11739
**15.6 µM PNA**	13237	13638	13955	7080	11682	14299	13472	10981
**15.6 µM PNA**	12133	13422	15298	6213	11320	14403	13553	10888
**15.6 µM PNA**	12880	14404	15366	6515	12129	13744	13865	11706
**15.6 µM PNA**	11502	14394	16174	6691	12688	14136	13635	11157
**15.6 µM PNA**	12863	12018	14775	6607	11544	14184	13323	11145
**15.6 µM PNA**	13705	13407	15744	6940	11985	14045	14372	11229
**31.3 µM PNA**	11180	11941	15038	5849	11058	13333	9395	13518
**31.3 µM PNA**	9492	10847	14025	5637	11272	13558	10171	13076
**31.3 µM PNA**	9943	14071	14767	5588	12997	13392	10733	13438
**31.3 µM PNA**	9194	13800	14783	5882	11658	13291	11165	13548
**31.3 µM PNA**	11500	12739	15232	6430	11316	13892	10212	14851
**31.3 µM PNA**	13171	13457	12104	5340	11091	13982	10222	12994
**62.5 µM PNA**	11899	13463	13575	5714	10729	13792	10327	11710
**62.5 µM PNA**	12100	12417	14760	5857	10929	14194	10234	12955
**62.5 µM PNA**	11451	12849	13975	5490	10364	14580	11042	13071
**62.5 µM PNA**	10997	12174	14171	5517	11246	12831	9873	13525
**62.5 µM PNA**	9615	9765	14756	5694	9892	13408	9724	12031
**62.5 µM PNA**	10609	12377	15678	5574	10970	13269	9990	13000
**Peanut butter protein fraction**	12527	11819	12432	7421	10917	13562	12805	10602
**Peanut butter protein fraction**	11817	13489	12447	6071	10939	14325	12742	11029
**Peanut butter protein fraction**	12431	12907	12290	6705	11614	13640	13685	10019
**Peanut butter protein fraction**	13131	13823	12982	5558	10810	13253	12630	10614
**Peanut butter protein fraction**	13146	13460	13387	6122	10565	12740	13003	10935
**Peanut butter protein fraction**	12956	12519	14073	6860	10949	13280	12110	10448
**Mixed nut butter protein fraction**	13509	15064	15987	8483	13345	15308	15054	12481
**Mixed nut butter protein fraction**	14552	15262	16774	8835	13297	14964	24327	12370
**Mixed nut butter protein fraction**	13898	15607	16750	10040	13258	14185	14380	12900
**Mixed nut butter protein fraction**	14156	14950	15693	10120	13608	14736	14873	12904
**Mixed nut butter protein fraction**	14728	16251	16254	8377	12588	14849	14567	12319

3.2.3 LDA results

Table S11 Classification of unknown analytes using LDA training set constructed using raw emission data. Concentrations of PNA are expressed as subunit concentration. The training set was constructed using a 125 µM concentration of PNA.

**Sample**	**LDA classification**	**Correct**
7.8 µM PNA (x 6)	PNA	6/6
15.6 µM PNA (x 6)	PNA	6/6
31.3 µM PNA (x 6)	PNA	6/6
62.5 µM PNA (x 6)	PNA	6/6
Peanut butter protein fraction (x 6)	PNA	6/6
Mixed nut butter protein fraction (x 6)	PNA	6/6

3.3 Using the reduced 4 sensor array

Table S12 Classification of unknown analytes using LDA training set constructed using four glycopolymer sensors. Concentrations of PNA are expressed as subunit concentration. The training set was constructed using a 125 µM concentration of PNA.

**Sample**	**LDA classification**	**Correct**
7.8 µM PNA (x 6)	PNA	6/6
15.6 µM PNA (x 6)	PNA	6/6
31.3 µM PNA (x 6)	PNA	6/6
62.5 µM PNA (x 6)	PNA	6/6
Peanut butter protein fraction (x 6)	PNA	6/6
Mixed nut butter protein fraction (x 6)	PNA	6/6

4. Discrimination of bacteria

4.1 Bacterial strain details

Enterococcus faecium (VSE)	DSM 13589
Enterococcus faecium (VRE)	DSM 17050
E. coli K-12 MG1655	Kindly provided by Dr Christoph Baumann, University of York
Salmonella enterica serovar typhimarium 2	Kindly provided by Dr Christoph Baumann, University of York
Pseudomonas aeruginosa PAO1	PAO1
Pseudomonas aeruginosa PA14	PA14

4.2 Discrimination using emission change ratio

4.2.1 Data

Table S13 Data used for LDA and construction of training set

Strain	P2NGal	P2NLac	P2NFuc	P2NNeu5Ac	P2NMan	P2NGlc	P2NGalNAc	P2NGlcNAc
**VRE**	1.04401281	1.03404747	1.17422604	1.32175772	1.05352706	1.02760902	1.03617598	1.01889369
**VRE**	1.05061	1.00833574	1.0760791	1.33762089	1.0243078	1.03535297	1.02954544	1.02125737
**VRE**	1.05549745	1.0129076	1.10011299	1.30862418	1.05034555	1.03382173	1.03270325	1.01234415
**VRE**	1.04996942	1.00663533	1.07356545	1.33013919	1.05602509	1.02594579	1.02929125	1.04050296
**VRE**	1.04995276	1.01684908	1.11133646	1.27949237	1.02098637	1.0087548	1.041466	1.0059146
**VSE**	1.00858732	0.97747999	1.02842082	1.26879768	1.04835433	0.99396228	1.00391642	1.02011646
**VSE**	1.00676025	0.98575598	1.03115355	1.42325188	1.01944905	1.01688043	0.99971363	0.99854167
**VSE**	1.02584176	0.99939977	1.11188005	1.14884154	1.01608056	0.98374504	0.99564597	1.0093122
**VSE**	1.02598929	0.99720762	1.06131346	1.21604772	1.05157877	1.03339328	0.9609929	1.00721672
**VSE**	1.00193713	1.01790557	1.08132059	1.22210078	1.03618436	1.00057933	1.00747913	1.003809
***E. coli* MG1665**	1.03973236	1.03973236	1.0816517	1.40578543	1.0455229	1.01355206	1.01620341	1.02183885
***E. coli* MG1665**	1.02659807	1.02659807	1.08554312	1.39250282	1.08328765	1.02210751	1.02241865	1.02000321
***E. coli* MG1665**	1.01574854	1.01574854	1.10039877	1.38572505	1.06257119	1.02067077	1.02608133	1.01833758
***E. coli* MG1665**	1.03675854	1.03675854	1.10027121	1.36464921	1.09029911	1.02201853	1.03450924	1.02271063
***E. coli* MG1665**	1.05109169	1.05109169	1.10200001	1.34670825	1.05725076	1.0158919	1.03034308	1.01880147
S. enterica	0.99730357	0.95760736	0.99172809	1.09342434	0.98949079	0.98552014	0.96318087	0.95989111
S. enterica	0.98374377	0.9506085	0.99979774	1.11066209	0.99093436	0.97433456	0.96925974	0.96711228
S. enterica	0.98738431	0.95789057	0.99372835	1.07725815	0.97812226	0.96632936	0.97080218	0.96079252
S. enterica	0.98382441	0.95335721	0.99426687	1.07420687	0.97532787	0.97196596	0.97506562	0.9522252
S. enterica	0.9805615	0.95822305	1.0012393	1.06800916	0.97888205	0.98009694	0.95954555	0.96769096
**PA01**	0.94738129	0.94964822	1.02087956	1.07500813	0.96779527	0.97200438	0.94555876	0.96782399
**PA01**	0.94072119	0.94715659	0.99127152	1.04657617	0.96972602	0.97798476	0.94816106	0.99909261
**PA01**	0.95057467	0.96589648	0.99705359	1.03225719	0.96128882	0.98966008	0.9496922	0.96283333

**PA01**	0.94625006	0.93544384	1.01321281	1.0831359	0.95488759	0.9581185	0.9283006	0.96253643
**PA01**	0.94471101	0.93318112	0.99949852	1.07106595	0.95792568	1.00598399	0.96203088	0.97123665
**PA14**	0.95319235	0.93352642	0.9987633	1.13452469	0.99629063	0.95522774	0.97178194	0.96016626
**PA14**	0.95030039	0.97211587	1.01801085	1.11426486	0.98106213	0.98023508	0.96906348	1.01323569
**PA14**	0.95767872	0.94092783	1.01392083	1.16654593	0.98756661	0.95357609	0.97320978	0.97132411
**PA14**	0.96422461	0.96293596	1.00325333	1.11212386	0.95523013	0.97169602	0.96905701	0.96686103
**PA14**	0.97647825	1.0015547	1.01291407	1.11616399	0.96243602	0.97064539	0.96709367	0.99781759
**buffer control**	0.92510287	0.92904501	0.92876712	0.93990796	0.92226703	0.93185291	0.93909107	0.92863679
**buffer control**	0.9360882	0.93835152	0.93423115	0.942314	0.91977058	0.942988	0.93475476	0.9434646
**buffer control**	0.93097074	0.9382452	0.93113157	0.95085353	0.93010805	0.93434958	0.93336317	0.92209402
**buffer control**	0.93143246	0.9416602	0.92743417	0.93786614	0.93082719	0.94107344	0.94033609	0.9276288
**buffer control**	0.93418066	0.94464649	0.93088583	0.94753893	0.93637365	0.93484231	0.93578043	0.95279349

4.2.3 LDA

Table S14 Canonical discriminant function coefficients for receptors obtained through LDA analysis

	Function				
	1	2	3	4	5
**P2NGal**	113.31	-71.194	-38.801	8.327	-40.398
**P2NLac**	-15.715	72.342	-32.763	-4.103	34.987
**P2NFuc**	27.544	-11.018	38.025	6.875	-4.514
**P2NNeu5Ac**	16.903	11.654	4.619	-1.53	-3.674
**P2NMan**	25.317	37.537	-39.009	28.502	-3.613
**P2NGlc**	9.762	-36.884	38.691	41.203	48.333
**P2NGalNAc**	60.585	-31.431	-5.546	-58.874	41.523
**P2NGlcNAc**	28.454	-8.497	50.732	-19.04	-55.096
**(Constant)**	-270.36	35.652	-18.346	-2.262	

Unstandardized coefficients

4.3 Reducing the array to 4 sensors

4.3.1 HCA analysis

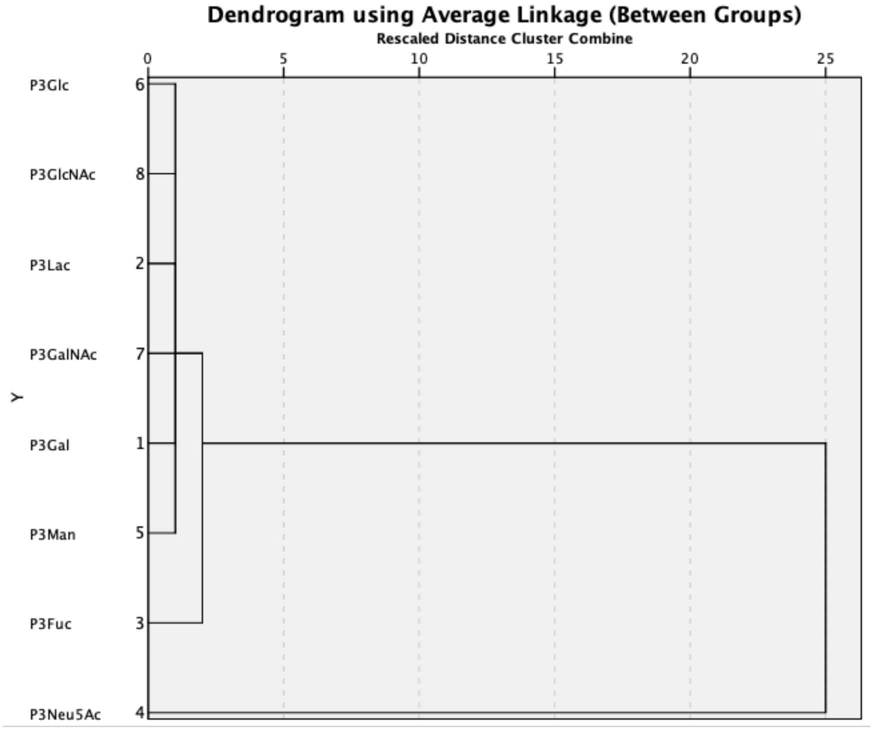

Fig. S11 Dendrogram produced through hierarchical cluster analysis (HCA) of glycopolymer receptor responses to addition of bacterial analytes.

4.3.2 Data

Table S15 Data used for LDA and construction of training set

Strain	P2NLac	P2NFuc	P2NNeu5Ac	P2NGalNAc
**VRE**	1.03404747	1.17422604	1.32175772	1.03617598
**VRE**	1.00833574	1.0760791	1.33762089	1.02954544
**VRE**	1.0129076	1.10011299	1.30862418	1.03270325
**VRE**	1.00663533	1.07356545	1.33013919	1.02929125
**VRE**	1.01684908	1.11133646	1.27949237	1.041466
**VSE**	0.97747999	1.02842082	1.26879768	1.00391642
**VSE**	0.98575598	1.03115355	1.42325188	0.99971363
**VSE**	0.99939977	1.11188005	1.14884154	0.99564597
**VSE**	0.99720762	1.06131346	1.21604772	0.9609929
**VSE**	1.01790557	1.08132059	1.22210078	1.00747913
**E coli MG1665**	1.03973236	1.0816517	1.40578543	1.01620341
**E coli MG1666**	1.02659807	1.08554312	1.39250282	1.02241865
**E coli MG1667**	1.01574854	1.10039877	1.38572505	1.02608133

**E coli MG1668**	1.03675854	1.10027121	1.36464921	1.03450924
**E coli MG1669**	1.05109169	1.10200001	1.34670825	1.03034308
**S. enterica**	0.95760736	0.99172809	1.09342434	0.96318087
**S. enterica**	0.9506085	0.99979774	1.11066209	0.96925974
**S. enterica**	0.95789057	0.99372835	1.07725815	0.97080218
**S. enterica**	0.95335721	0.99426687	1.07420687	0.97506562
**S. enterica**	0.95822305	1.0012393	1.06800916	0.95954555
**PA01**	0.94964822	1.02087956	1.07500813	0.94555876
**PA01**	0.94715659	0.99127152	1.04657617	0.94816106
**PA01**	0.96589648	0.99705359	1.03225719	0.9496922
**PA01**	0.93544384	1.01321281	1.0831359	0.9283006
**PA01**	0.93318112	0.99949852	1.07106595	0.96203088
**PA14**	0.93352642	0.9987633	1.13452469	0.97178194
**PA14**	0.97211587	1.01801085	1.11426486	0.96906348
**PA14**	0.94092783	1.01392083	1.16654593	0.97320978
**PA14**	0.96293596	1.00325333	1.11212386	0.96905701
**PA14**	1.0015547	1.01291407	1.11616399	0.96709367

4.3.3 LDA

Table S16 Canonical discriminant function coefficients for receptors obtained through LDA analysis

	Function			
	**1**	**2**	**3**	**4**
**P3-Lac**	31.035	28.83	-43.007	40.765
**P3-Fuc**	22.563	1.352	46.129	3.703
**P3-Neu5Ac**	18.551	13.132	2.183	-10.822
**P3-GalNAc**	46.804	-82.289	-28.339	-6.205
**(Constant)**	-122.816	35.811	19.539	-24.847

Unstandardized coefficients

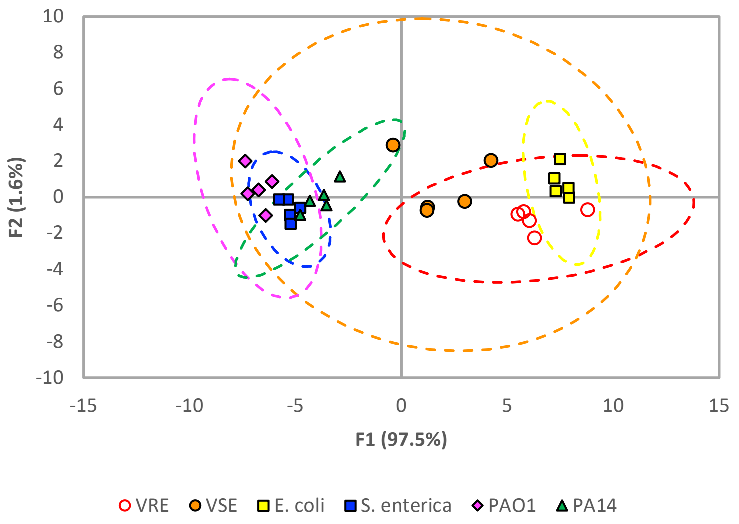

Fig. S12 Classification of analytes using LDA training set constructed using four glycopolymer sensors. Dashed lines denote 95% confidence intervals.

5 NMR spectra

2

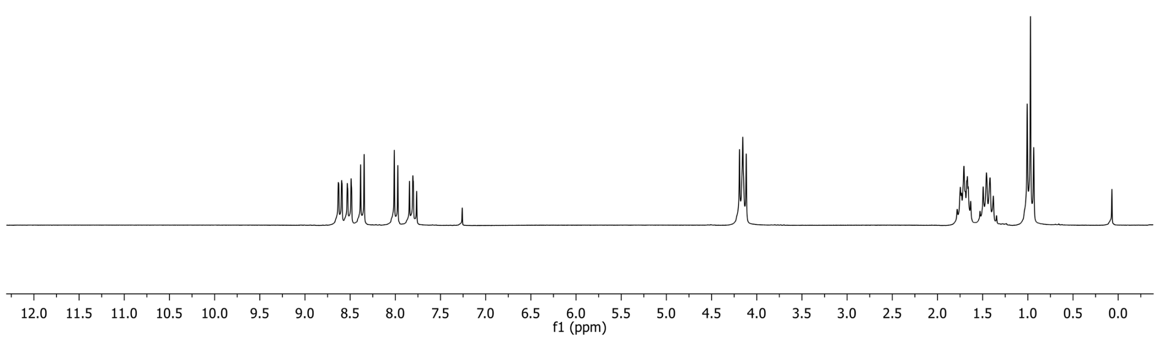

Fig. S13 ^{1}H NMR spectrum (300 MHz, $CDCl_3$) of *N*-butyl-4-bromo-1,8-naphthalimide **(2)**

1

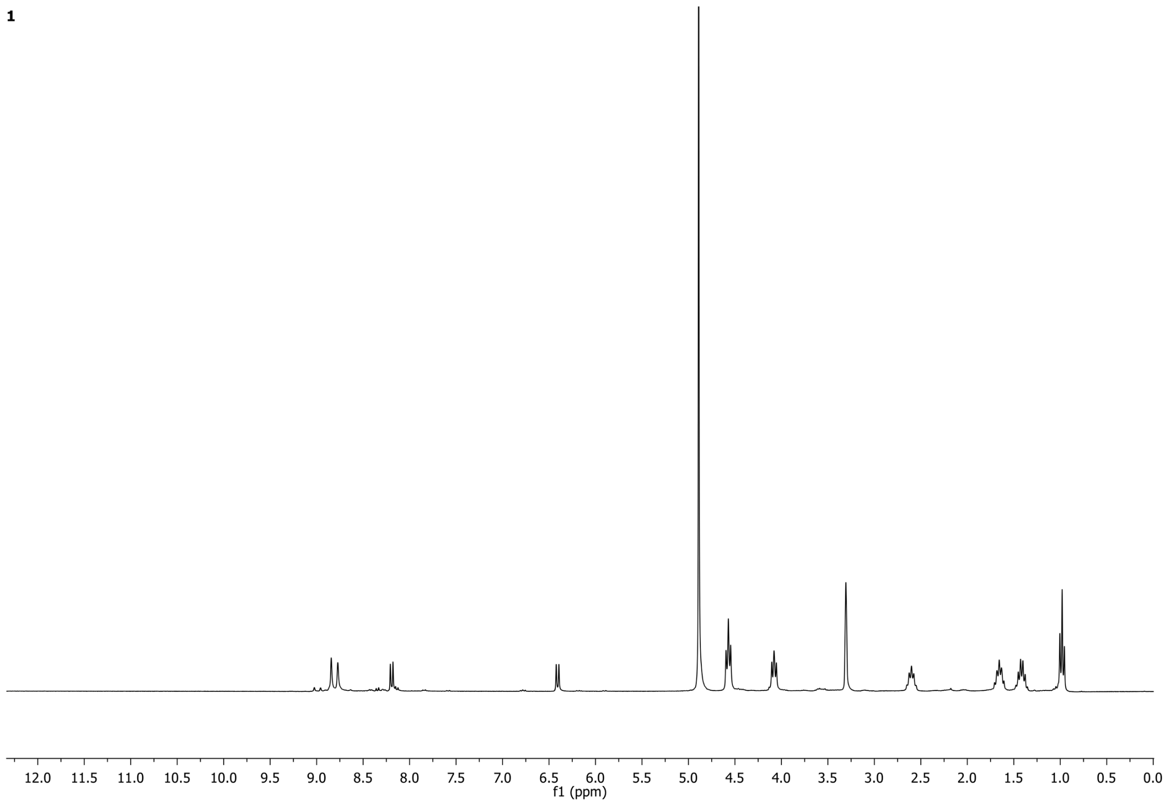

Fig. S14 ^{1}H NMR spectrum (300 MHz, MeOH-d_4) of *N*-butyl-4-propylbromo-6-sulfo-1,8-naphthalimide (**1**)

1

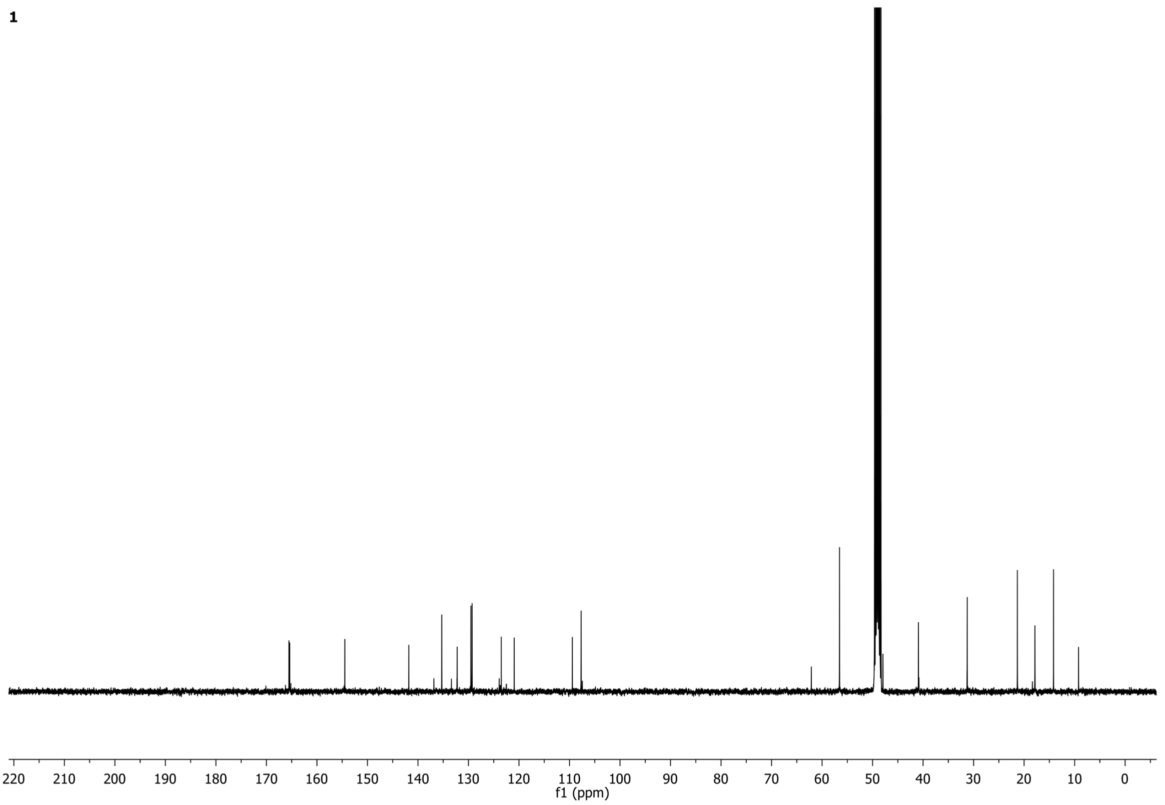

Fig. S15 ^{13}C NMR spectrum (100 MHz, MeOH-d_4) of *N*-butyl-4-propylbromo-6-sulfo-1,8-naphthalimide (**1**)

3

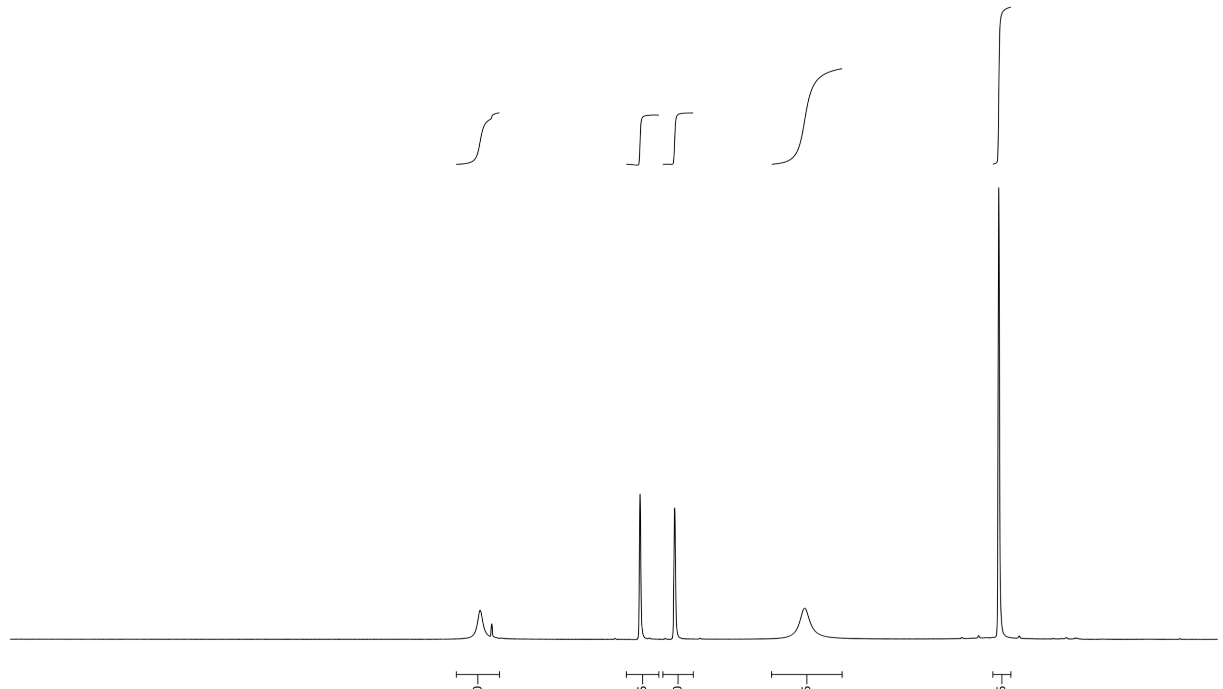

Fig. S16 ^{1}H NMR spectrum (300 MHz, $CDCl_3$) of methacryloyl hydrazide **3**

3

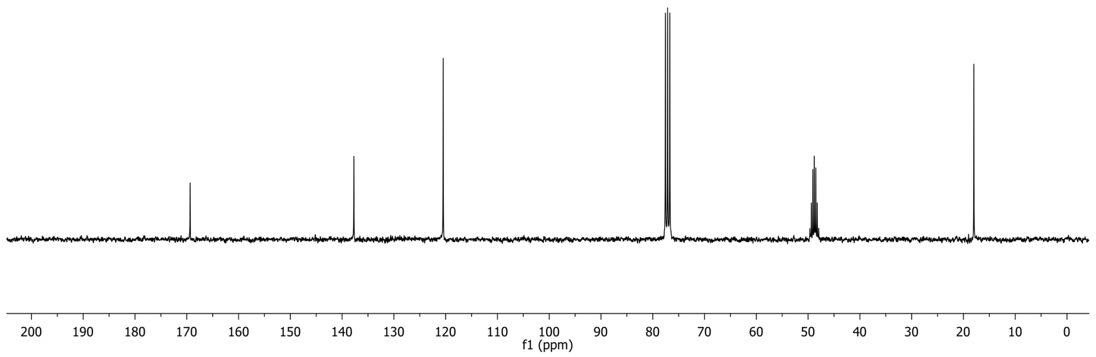

Fig. S17 ^{13}C NMR spectrum (75 MHz, $CDCl_3$/MeOH-d_4) of methacryloyl hydrazide **3**

M1

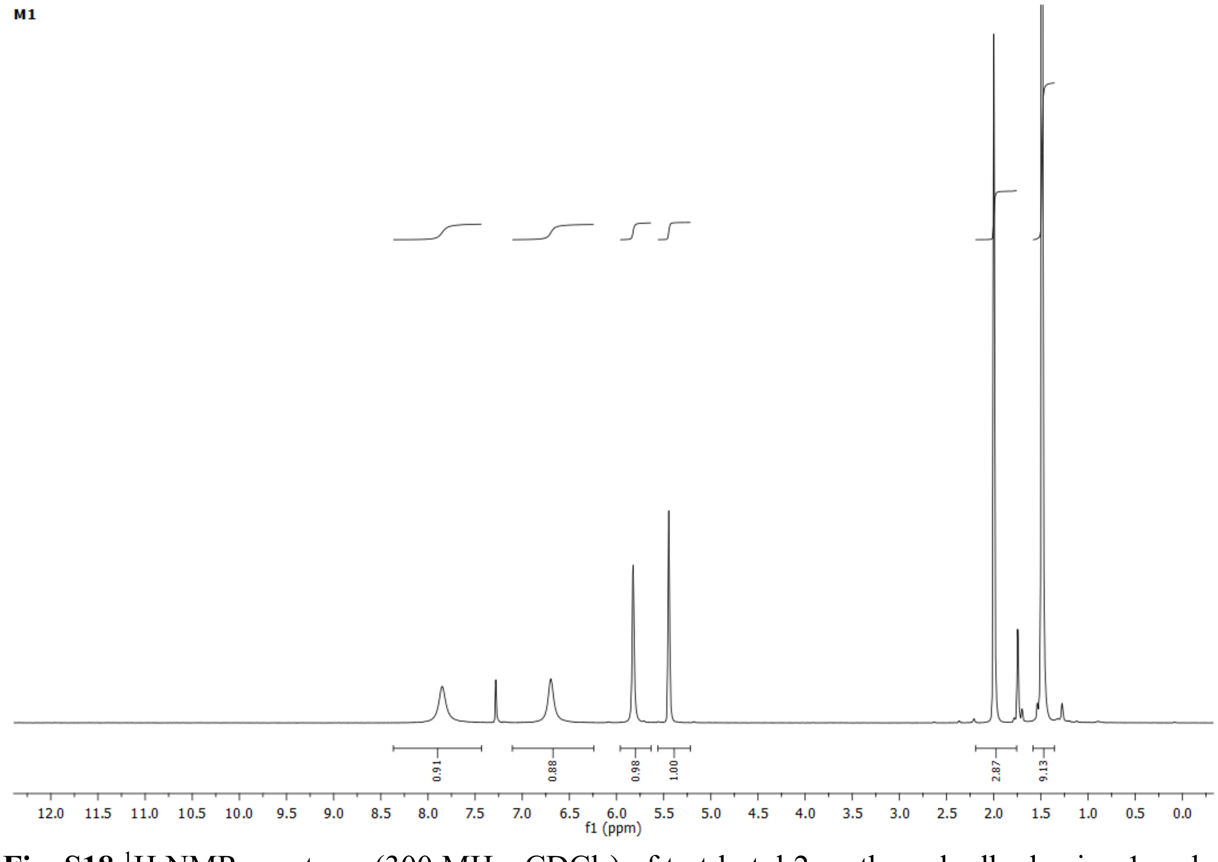

Fig. S18 ^{1}H NMR spectrum (300 MHz, $CDCl_3$) of tert-butyl 2-methacryloylhydrazine-1-carboxylate (**M1**)

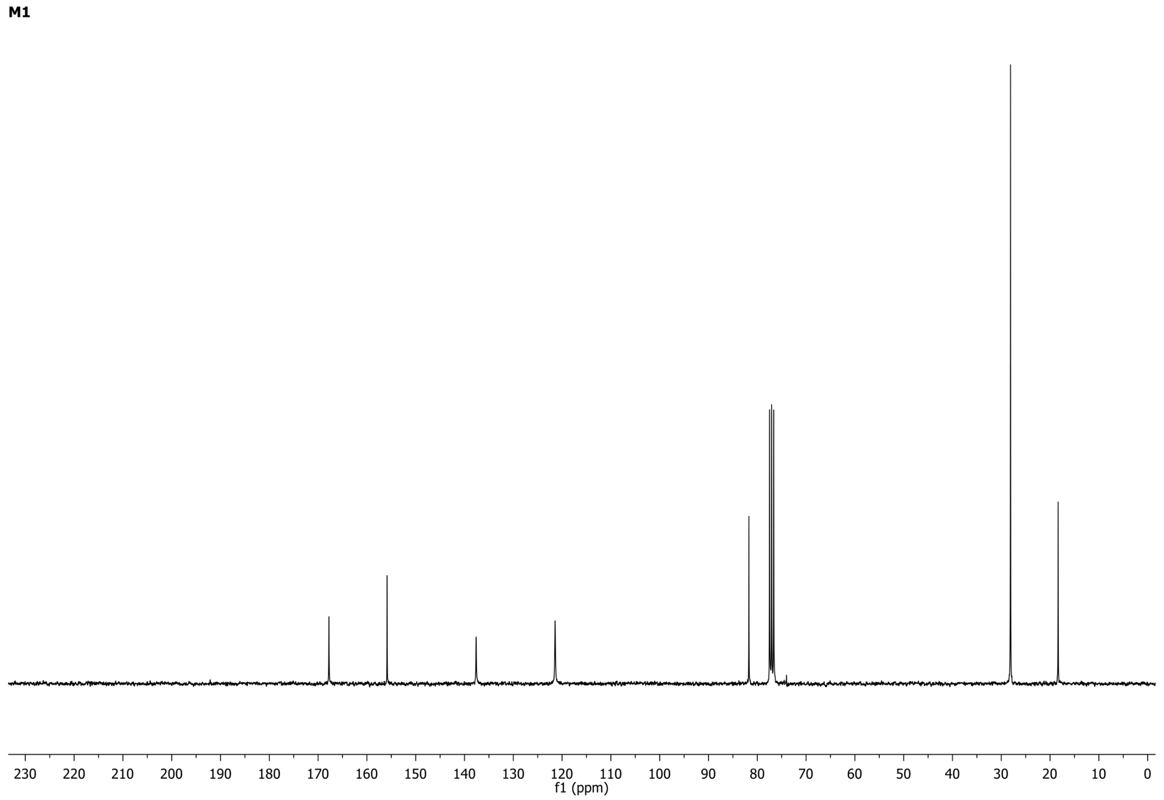

Fig. S19 ^{13}C NMR spectrum (75 MHz, $CDCl_3$) of tert-butyl 2-methacryloylhydrazine-1-carboxylate (**M1**)

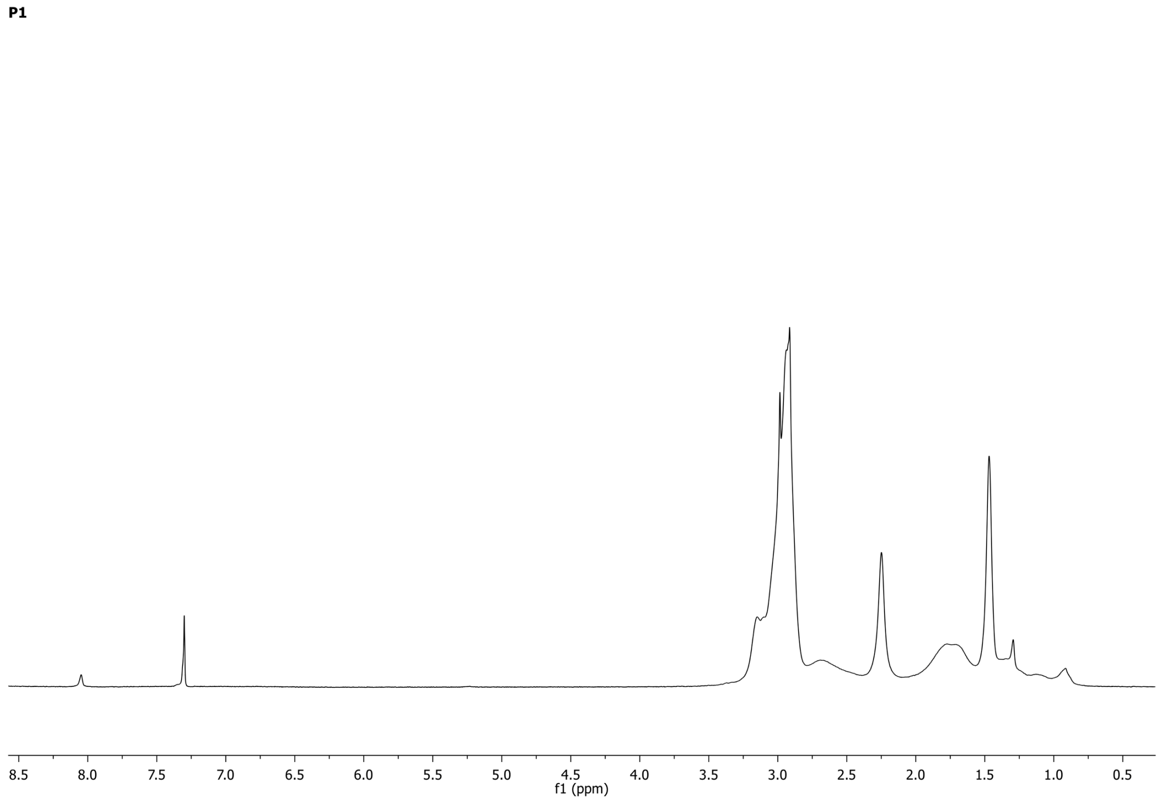

Fig. S20 ^{1}H NMR spectrum (300 MHz, $CDCl_3$) of **P1**

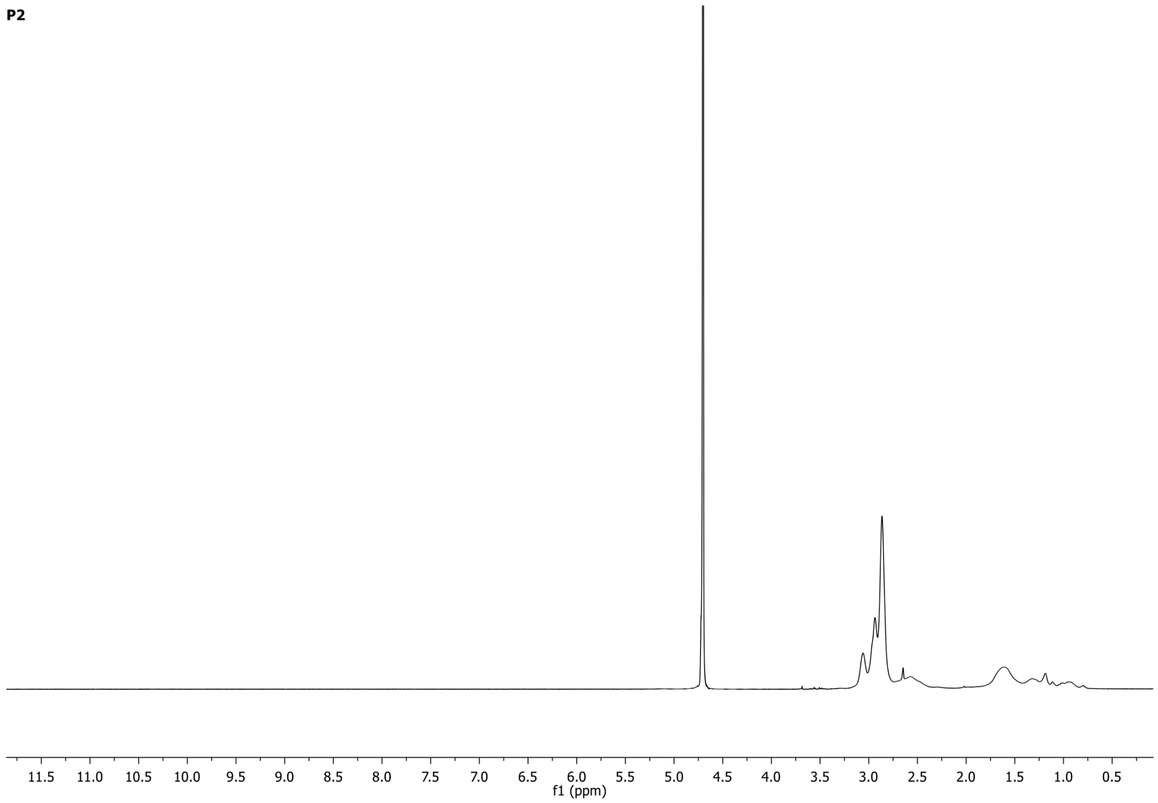

Fig. S21 ^{1}H NMR spectrum (300 MHz, D_2O) of **P2**

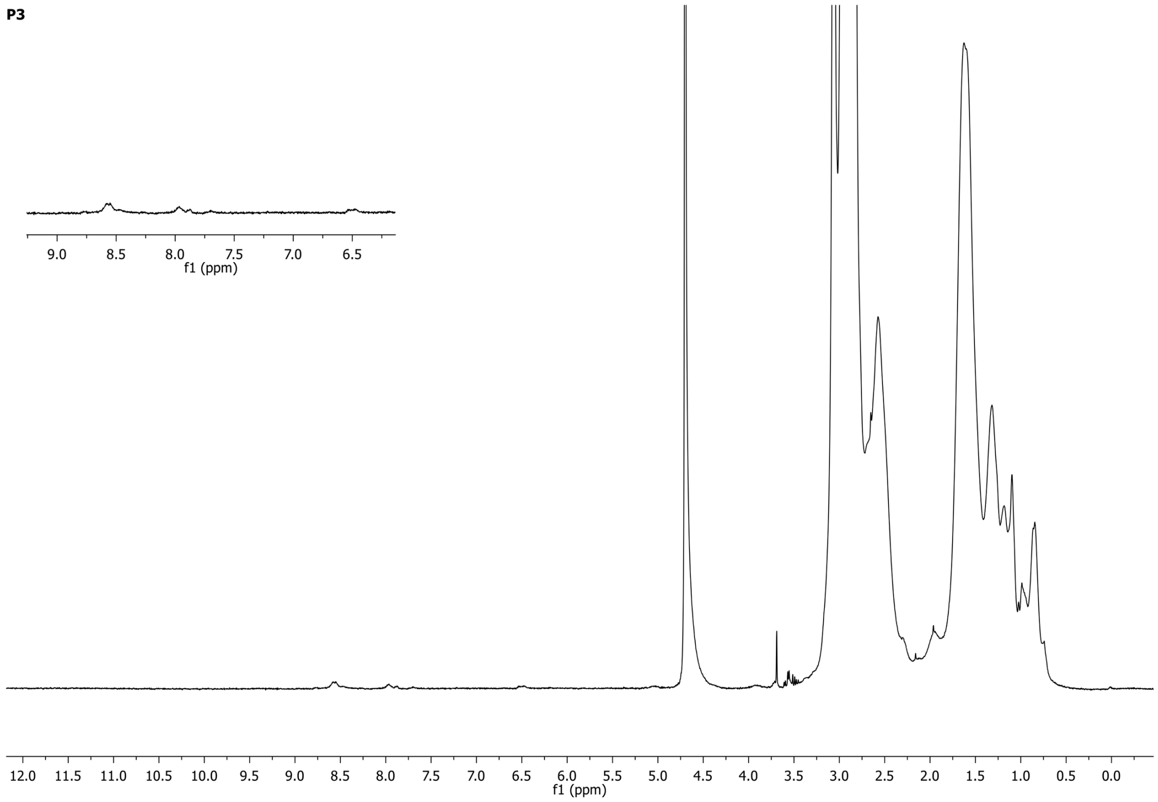

Fig. S22 ^{1}H NMR spectrum (300 MHz, D_2O) of **P3**

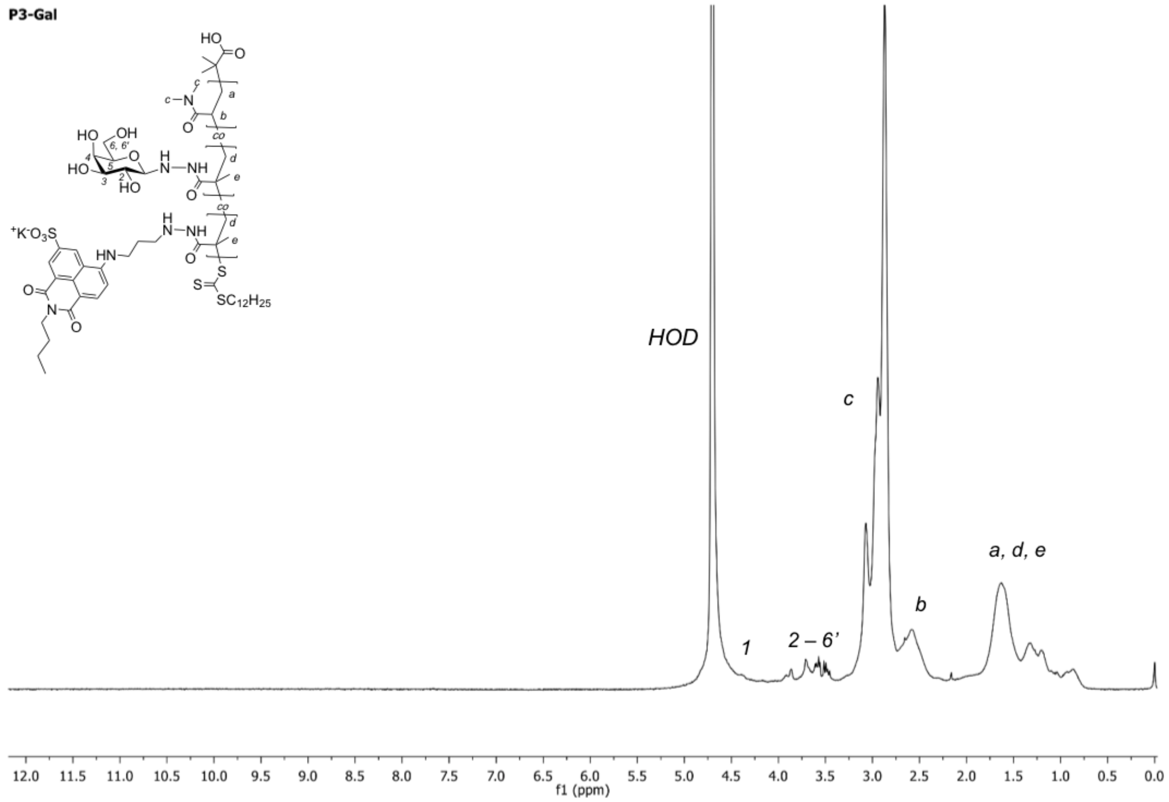

Fig. S23 ^{1}H NMR spectrum (300 MHz, D_2O) of **P3-Gal**

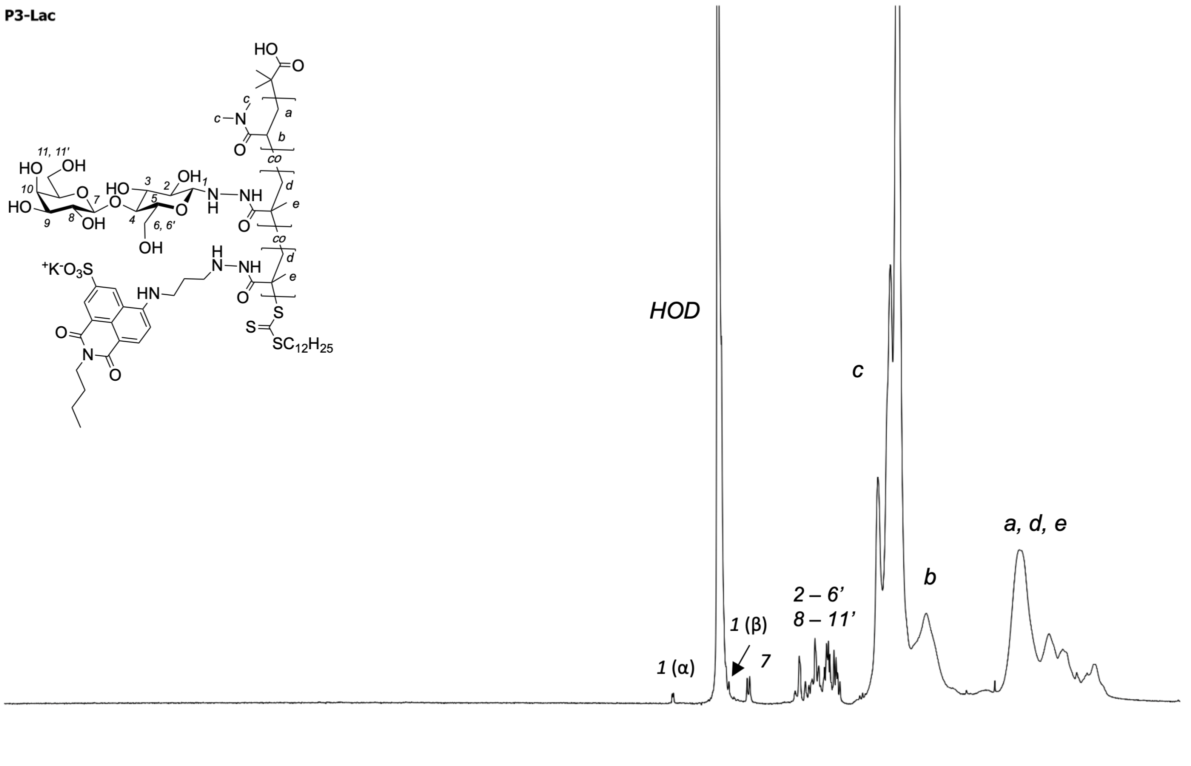

Fig. S24 ^{1}H NMR spectrum (300 MHz, D_2O) of **P3-Lac**. Integration indicates ~75% β anomer present.

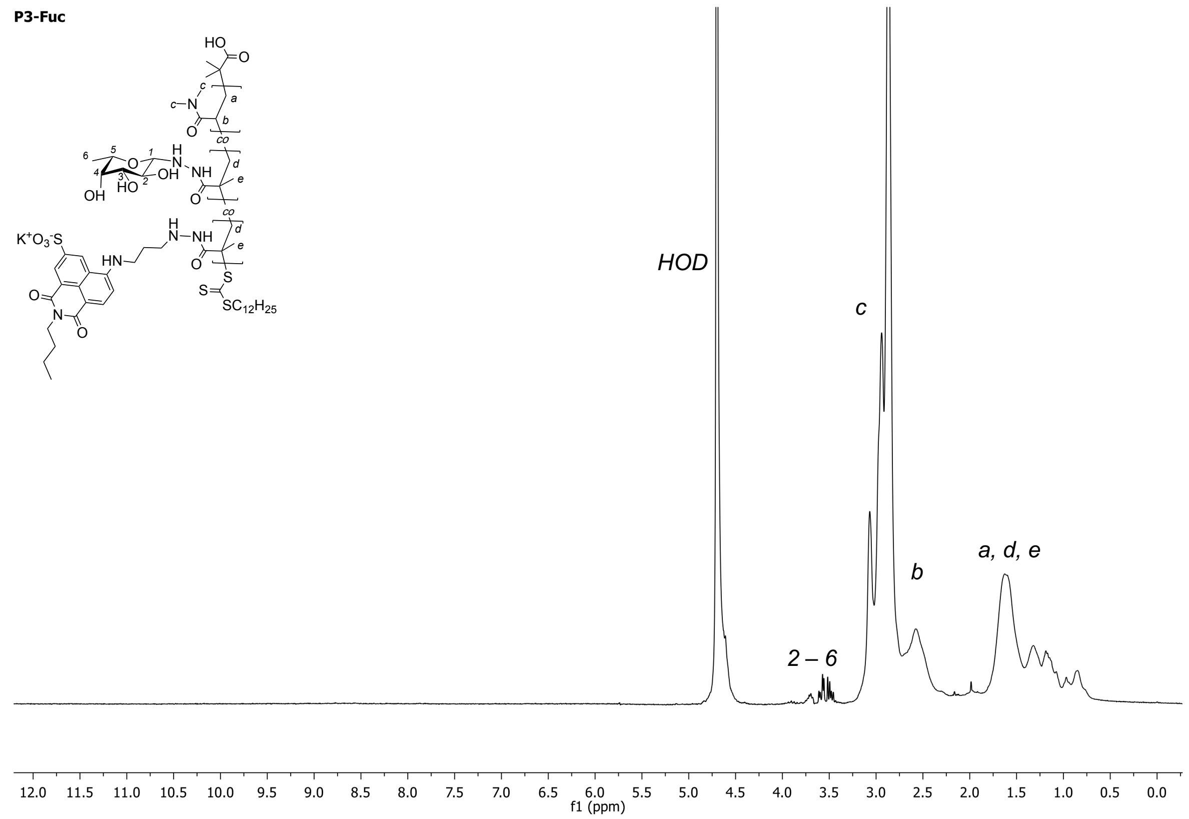

Fig. S25 ^{1}H NMR spectrum (300 MHz, D_2O) of **P3-Fuc**

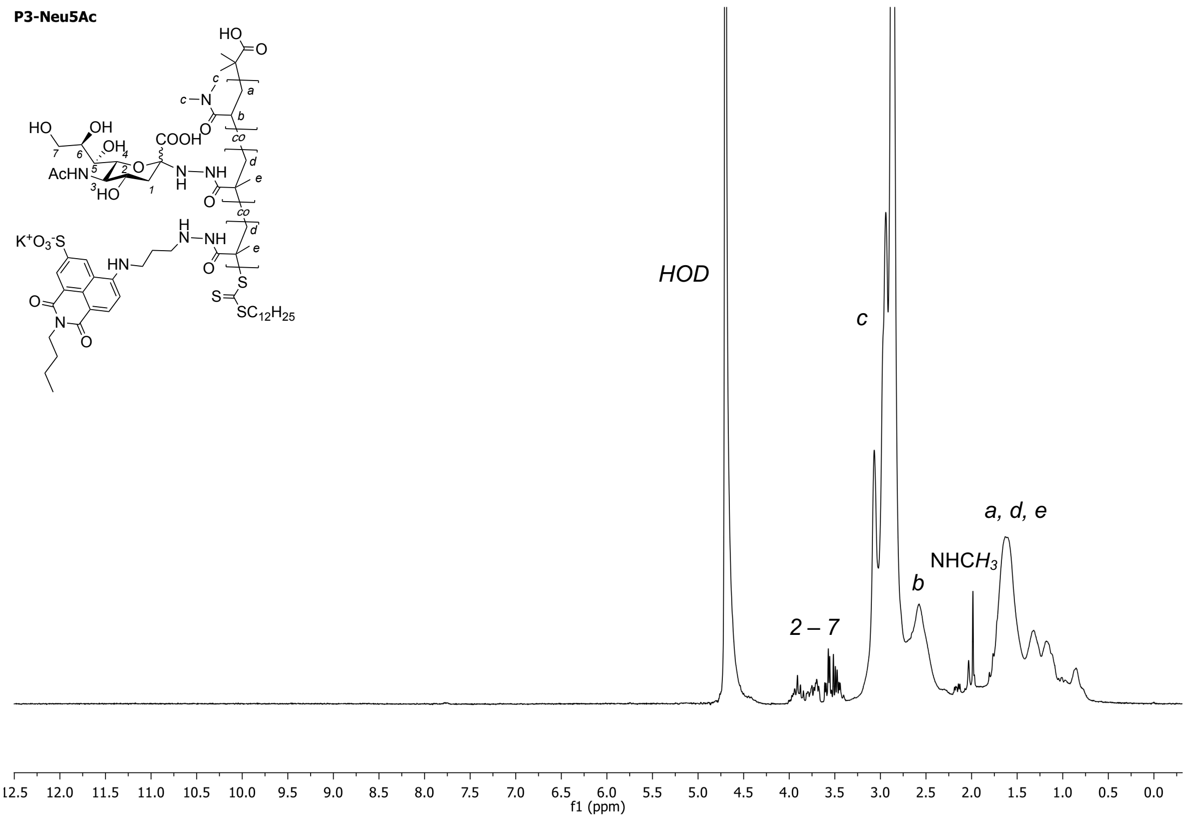

Fig. S26 ^{1}H NMR spectrum (300 MHz, D_2O) of **P3-Neu5Ac**

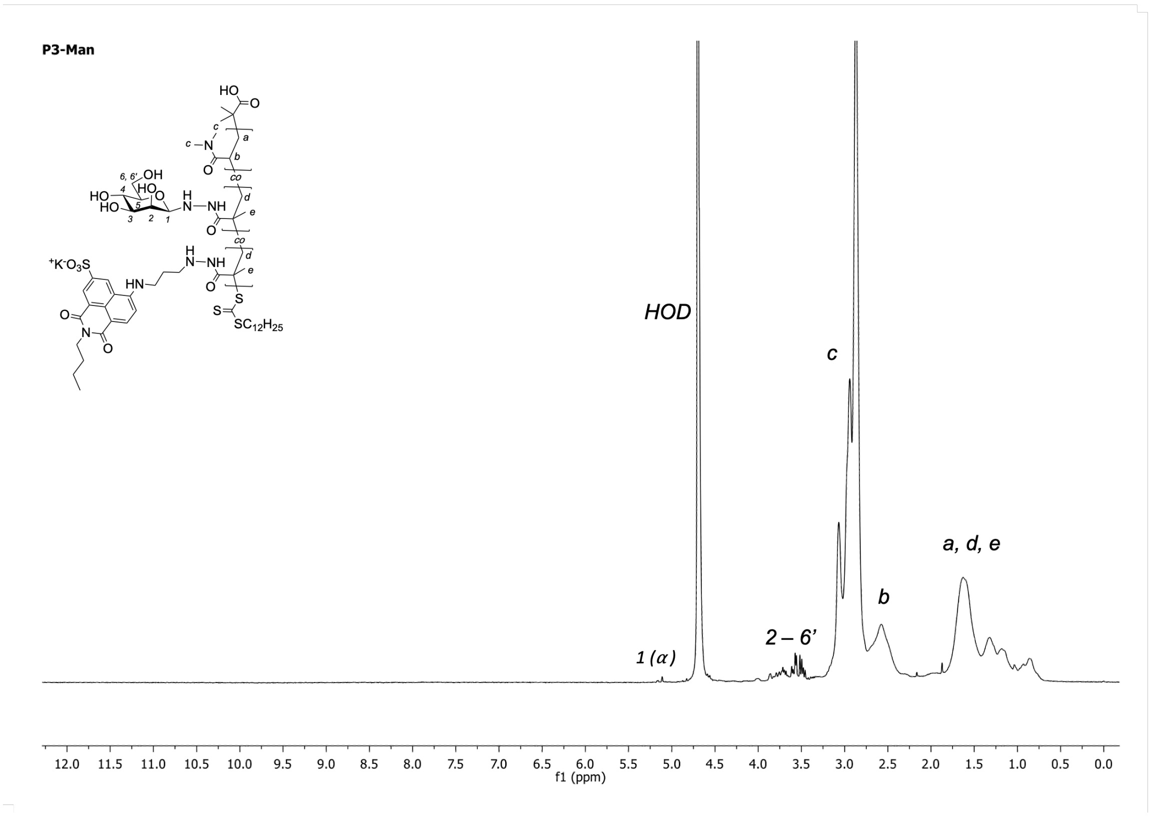

Fig. S27 ^{1}H NMR spectrum (300 MHz, D_2O) of **P3-Man.** Integration indicates ~85% β anomer present.

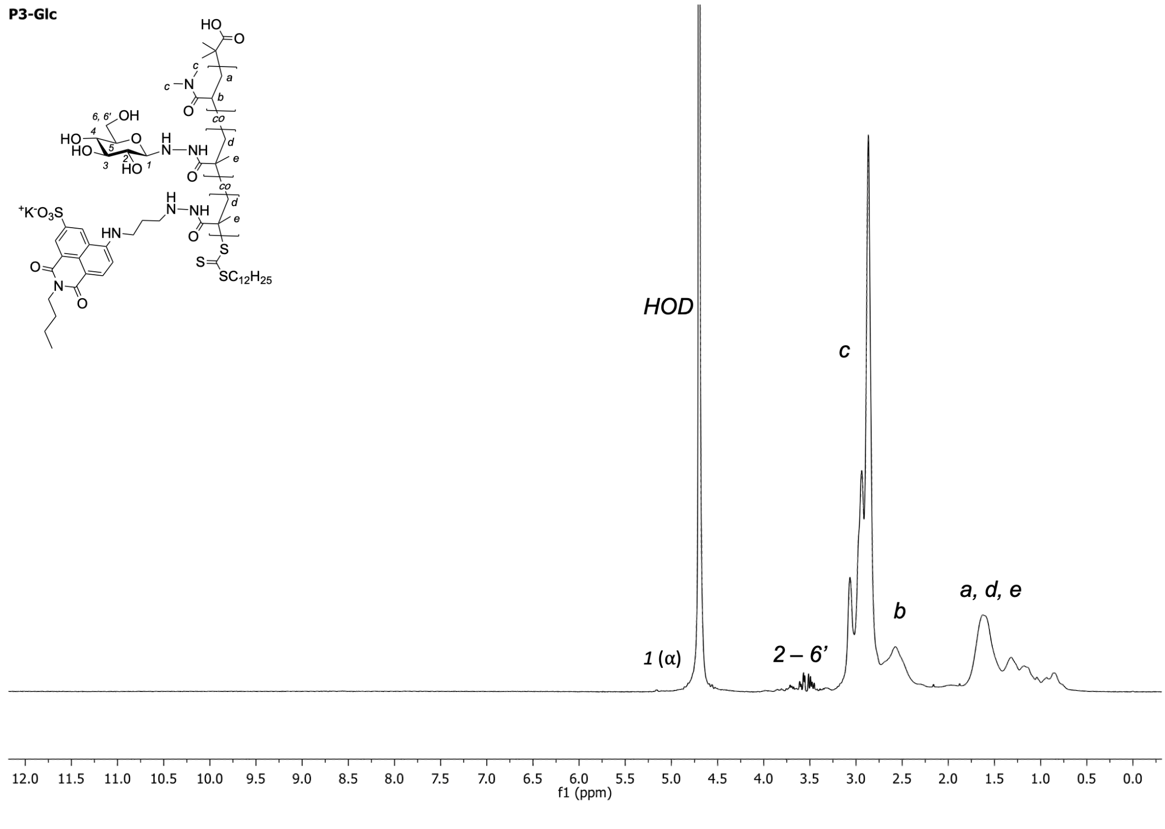

Fig. S28 ^{1}H NMR spectrum (300 MHz, D_2O) of **P3-Glc.** Integration indicates ~85% β anomer present.

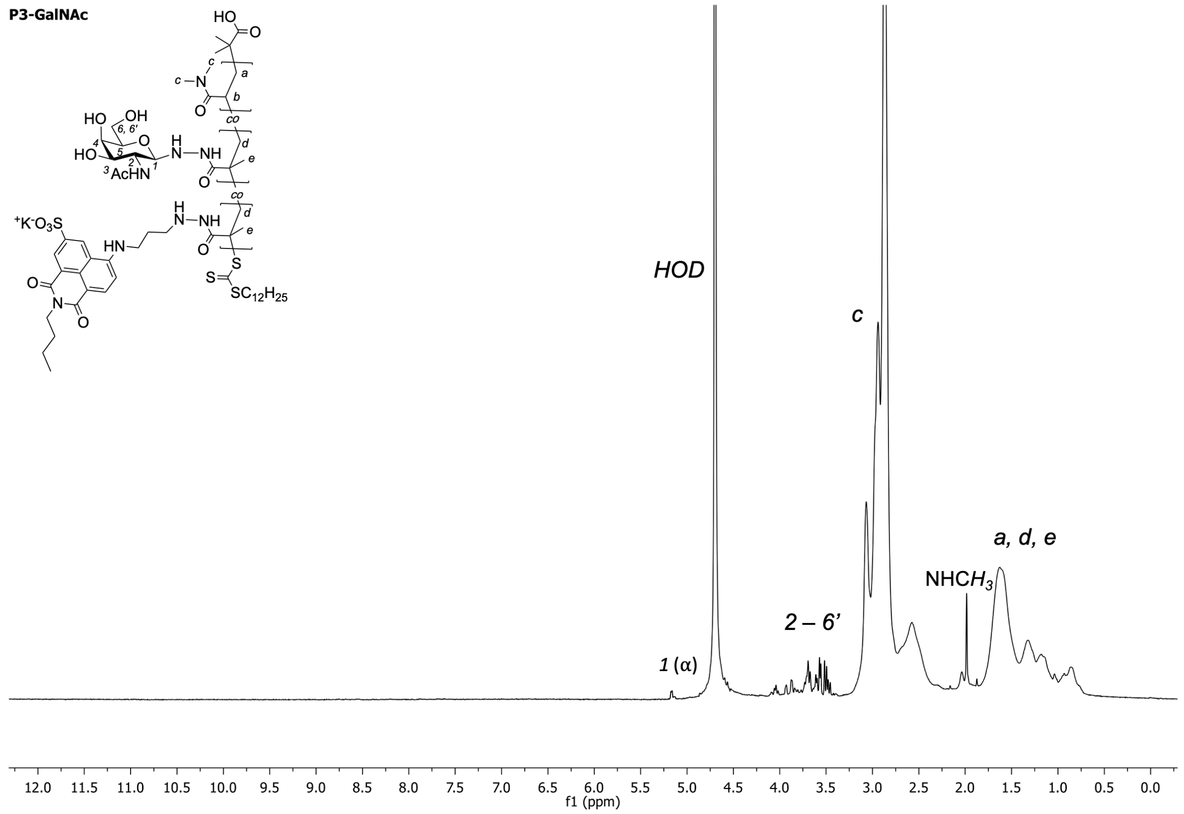

Fig. S29 ^{1}H NMR spectrum (300 MHz, D_2O) of **P3-GalNAc.** Integration indicates ~70% β anomer present.

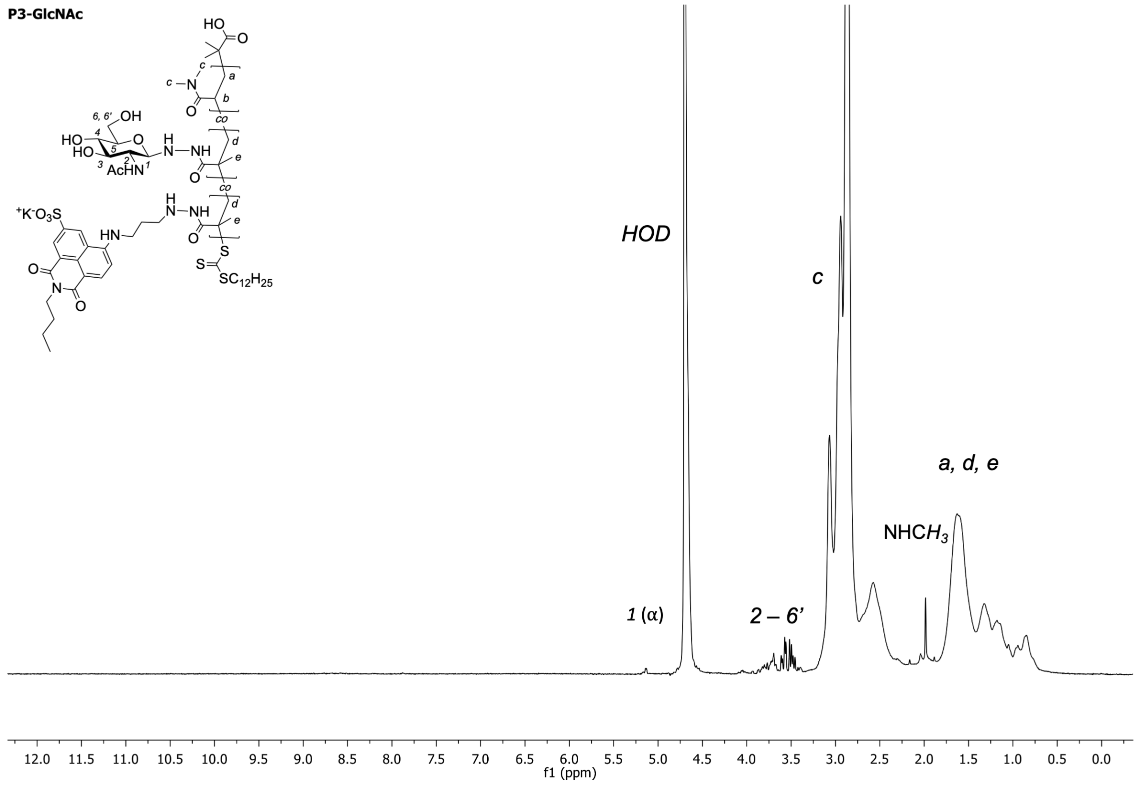

Fig. S30 ^{1}H NMR spectrum (300 MHz, D_2O) of **P3-GlcNAc.** Integration indicates ~75% β anomer present.

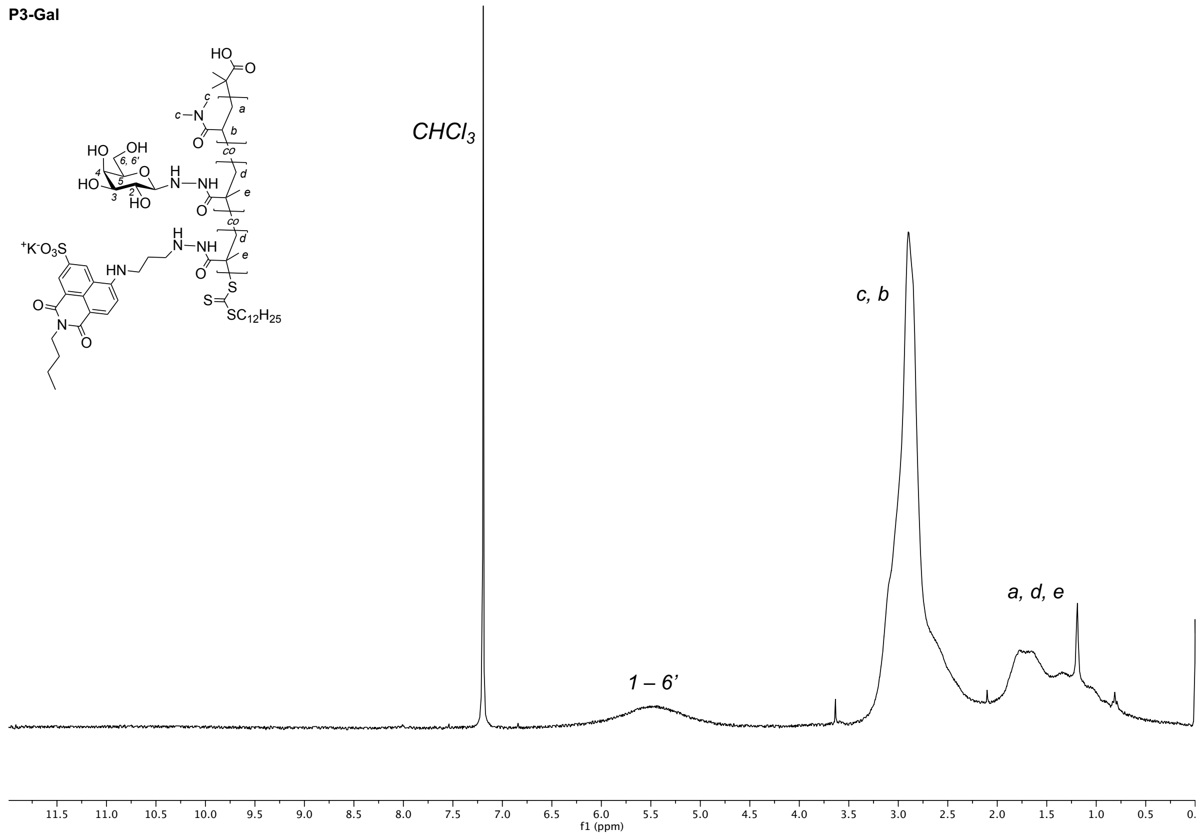

Fig. S31 ^{1}H NMR spectrum (300 MHz, $CDCl_3$) of **P3-Gal**

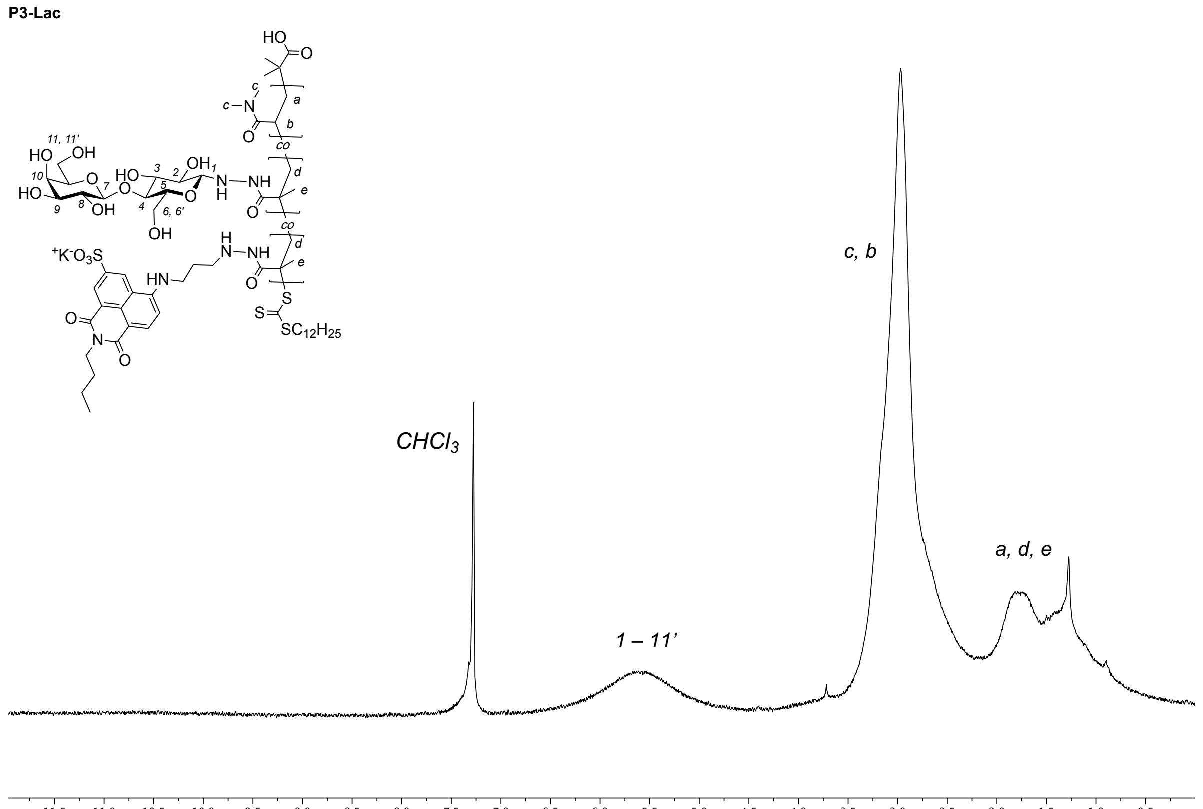

Fig. S32 ^{1}H NMR spectrum (300 MHz, $CDCl_3$) of **P3-Lac**

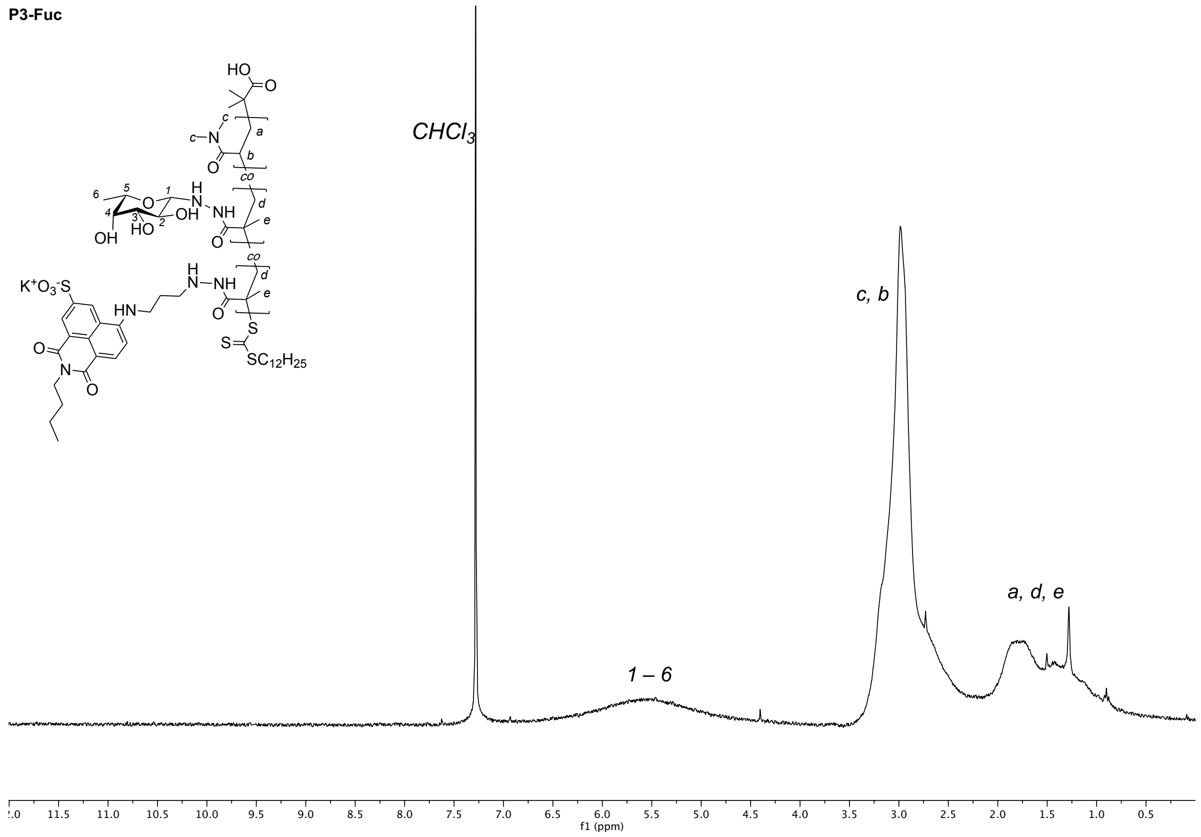

Fig. S33 ^{1}H NMR spectrum (300 MHz, $CDCl_3$) of **P3-Fuc**

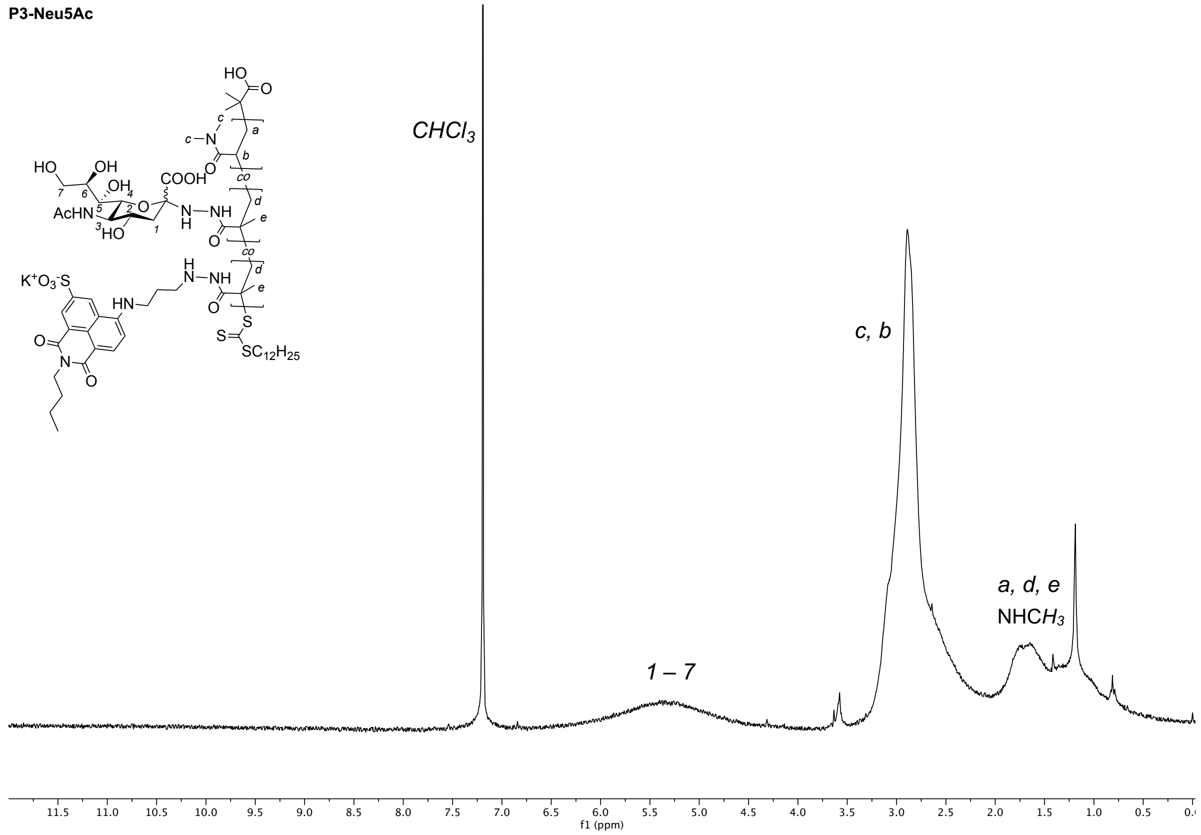

Fig.. S34 ^{1}H NMR spectrum (300 MHz, $CDCl_3$) of **P3-Neu5Ac**

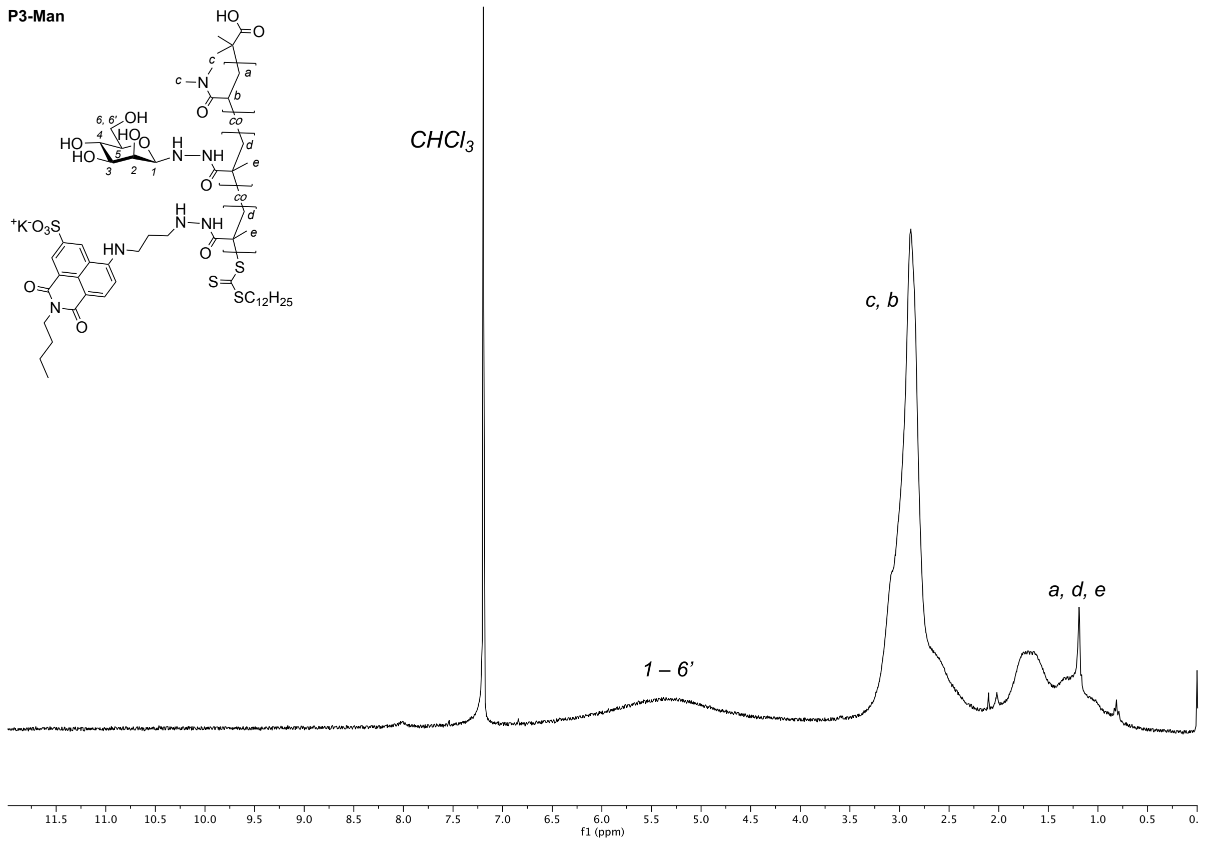

Fig. S35 ^{1}H NMR spectrum (300 MHz, $CDCl_3$) of **P3-Man**

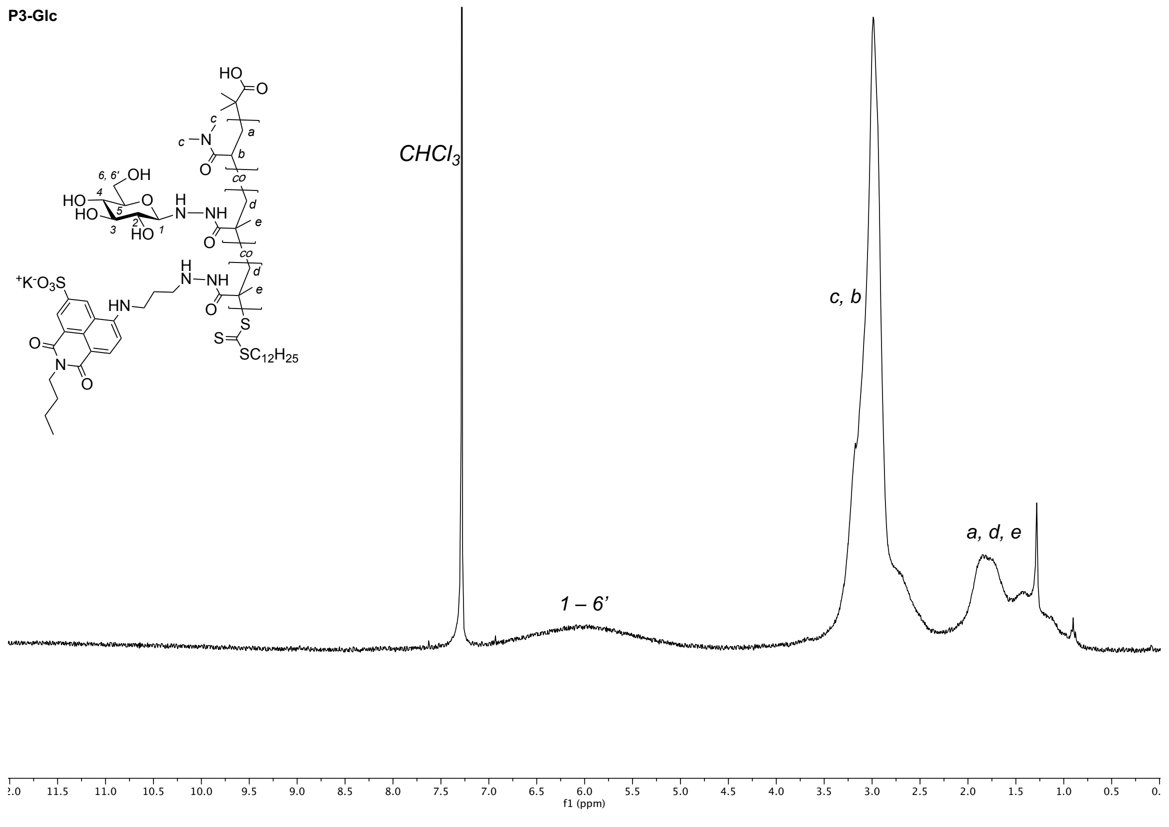

Fig. S36 ^{1}H NMR spectrum (300 MHz, $CDCl_3$) of **P3-Glc**

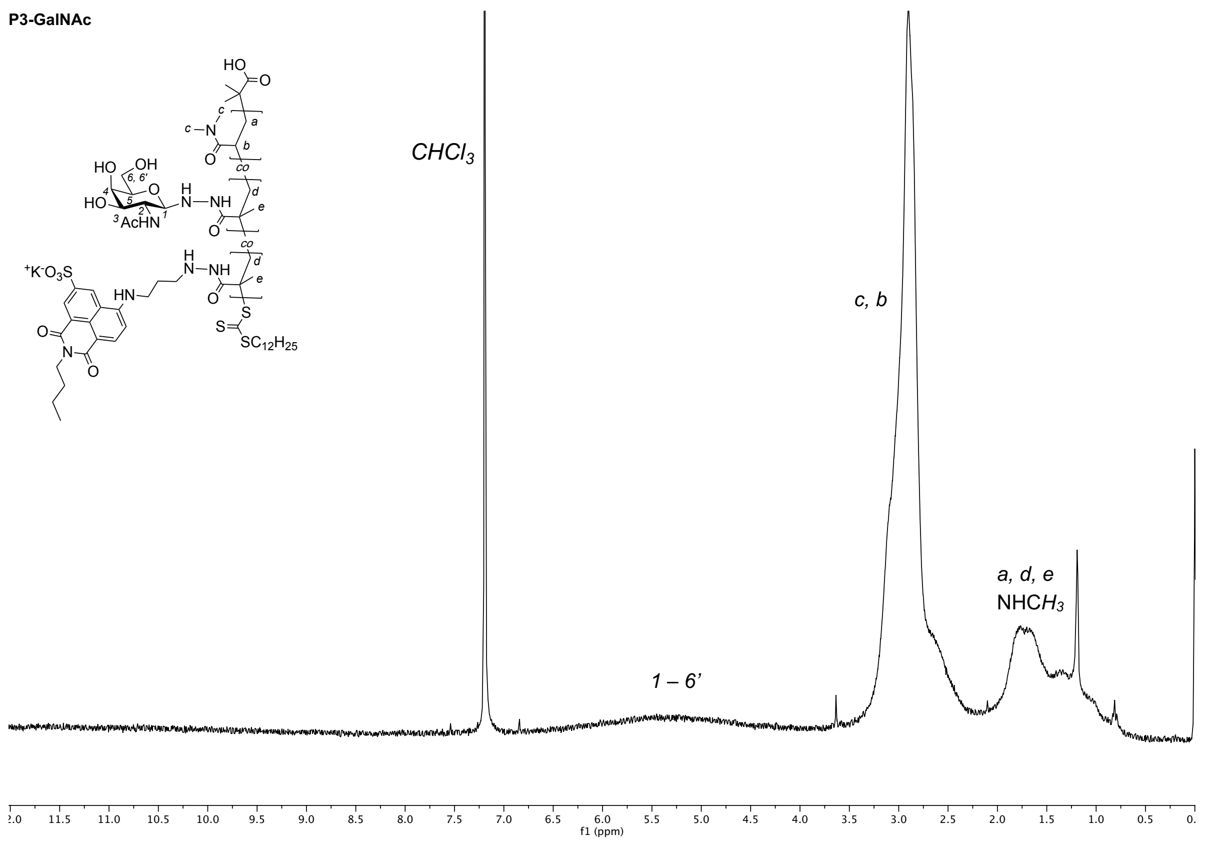

Fig. S37 ^{1}H NMR spectrum (300 MHz, $CDCl_3$) of **P3-GalNAc**

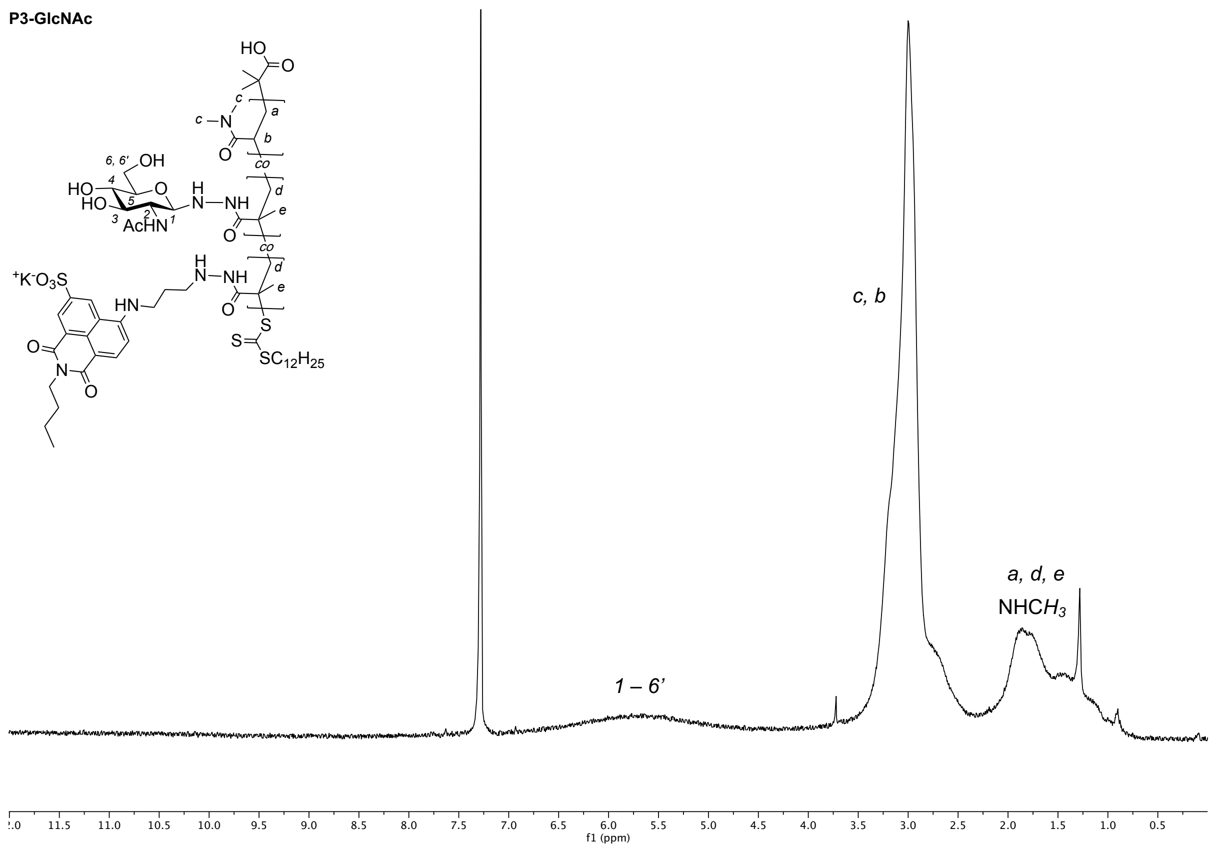

Fig. S38 ^{1}H NMR spectrum (300 MHz, $CDCl_3$) of **P3-GlcNAc**

6 References

1. Chen, Y.; Ballard, N.; Coleman, O. D.; Hands-Portman, I. J.; Bon, S. A. F., Dynamic control of volume phase transitions of poly(N-isopropylacrylamide) based microgels in water using hydrazide-aldehyde chemistry. *J. Polym. Sci. A Polym. Chem.,* **2014**, *52* (12), 1745-1754.
2. Mahon, C. S.; Wildsmith, G. C.; Haksar, D.; de Poel, E.; Beekman, J. M.; Pieters, R. J.; Webb, M. E.; Turnbull, W. B., A 'catch-and-release' receptor for the cholera toxin. *Faraday Discuss.,* **2019**, *219* (0), 112-117.
3. Mann, D. A.; Kanai, M.; Maly, D. J.; Kiessling, L. L., Probing Low Affinity and Multivalent Interactions with Surface Plasmon Resonance: Ligands for Concanavalin A. *Journal of the American Chemical Society,* **1998**, *120* (41), 10575-10582.
4. Neurohr, K. J.; Young, N. M.; Mantsch, H. H., Determination of the carbohydrate-binding properties of peanut agglutinin by ultraviolet difference spectroscopy. *J. Biol.Chem.,* **1980**, *255* (19), 9205-9209.
5. Merritt, E. A.; Sixma, T. K.; Kalk, K. H.; van Zanten, B. A. M.; Hol, W. G. J., Galactose-binding site in Escherichia coli heat-labile enterotoxin (LT) and cholera toxin (CT). *Mol. Microbiol.,* **1994**, *13* (4), 745-753.
6. Holmner, Å.; Mackenzie, A.; Ökvist, M.; Jansson, L.; Lebens, M.; Teneberg, S.; Krengel, U., Crystal Structures Exploring the Origins of the Broader Specificity of Escherichia coli Heat-Labile Enterotoxin Compared to Cholera Toxin. *J. Mol. Biol.,* **2011**, *406* (3), 387-402.
7. Turnbull, W. B.; Precious, B. L.; Homans, S. W., Dissecting the Cholera Toxin−Ganglioside GM1 Interaction by Isothermal Titration Calorimetry. *J. Am. Chem. Soc.,* **2004**, *126* (4), 1047-1054.
8. Lienemann, M.; Paananen, A.; Boer, H.; de la Fuente, J. M.; García, I.; Penadés, S.; Koivula, A., Characterization of the wheat germ agglutinin binding to self-assembled monolayers of neoglycoconjugates by AFM and SPR. *Glycobiology,* **2009**, *19* (6), 633-643.
9. Monsigny, M.; Roche, A.-C.; Sene, C.; Maget-Dana, R.; Delmotte, F., Sugar-Lectin Interactions: How Does Wheat-Germ Agglutinin Bind Sialoglycoconjugates? *Eur. J. Biochem.,* **1980**, *104* (1), 147-153.
10. Cohlberg, J. A.; Li, J.; Uversky, V. N.; Fink, A. L., Heparin and Other Glycosaminoglycans Stimulate the Formation of Amyloid Fibrils from α-Synuclein in Vitro. *Biochemistry,* **2002**, *41* (5), 1502-1511.
11. Gupta, D.; Cho, M.; Cummings, R. D.; Brewer, C. F., Thermodynamics of Carbohydrate Binding to Galectin-1 from Chinese Hamster Ovary Cells and Two Mutants. A Comparison with Four Galactose-Specific Plant Lectins. *Biochemistry,* **1996**, *35* (48), 15236-15243.

7 Appendix

Step by step guide on the use of LDA analysis with SPSS software.

1. Input data into a new spreadsheet in SPSS. Each analyte must be assigned to a category (1 – n) for training the model.

	Lectin	P3Gal	P3Lac	P3Fuc	P3Neu5Ac	P3Man	P3Glc	P3GalNAc	P3GlcNac
19	4.000	.874	.770	.794	.681	.721	.771	.783	.789
20	4.000	.872	.783	.767	.684	.722	.769	.783	.781
21	4.000	.885	.786	.763	.686	.754	.775	.809	.784
22	4.000	.864	.785	.827	.663	.723	.745	.773	.778
23	4.000	.866	.760	.820	.700	.762	.765	.780	.733
24	4.000	.886	.787	.802	.670	.728	.785	.776	.762
25	5.000	.706	.712	.741	.682	.666	.684	.690	.648
26	5.000	.689	.716	.918	.669	.687	.693	.742	.666
27	5.000	.718	.703	.731	.714	.687	.664	.690	.680
28	5.000	.725	.731	.756	.711	.668	.706	.703	.713
29	5.000	.712	.741	.698	.732	.688	.690	.693	.721
30	5.000	.639	.688	.655	.705	.698	.725	.694	.734
31	6.000	2.742	2.595	2.713	4.181	3.439	3.523	3.168	3.541
32	6.000	2.546	2.983	2.984	3.897	3.448	3.723	3.341	3.399
33	6.000	2.934	2.818	2.992	4.031	3.843	3.682	3.401	3.598
34	6.000	3.176	3.206	3.265	4.424	3.689	3.498	3.340	3.229
35	6.000	3.144	2.850	3.130	4.049	3.674	3.510	3.376	3.272
36	6.000	2.857	2.964	3.253	4.368	3.696	3.492	3.521	3.363

2. Define analyte categories as a ‘nominal’ variable and the fluorescence responses of each glycopolymer as ‘scale’ variables within the variable view tab.

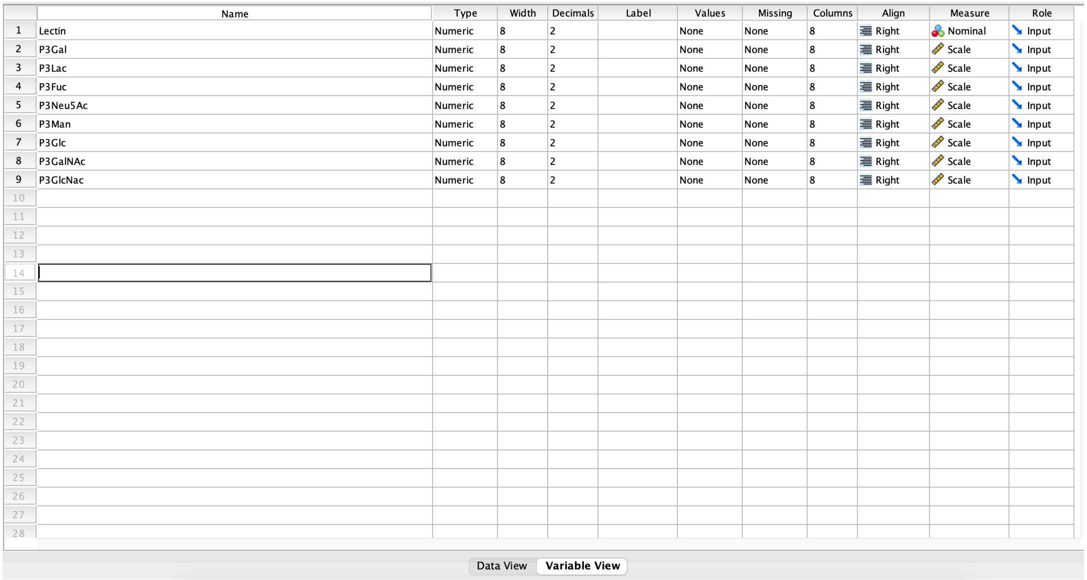

	Name	Type	Width	Decimals	Label	Values	Missing	Columns	Align	Measure	Role
1	Lectin	Numeric	8	2		None	None	8	Right	Nominal	Input
2	P3Gal	Numeric	8	2		None	None	8	Right	Scale	Input
3	P3Lac	Numeric	8	2		None	None	8	Right	Scale	Input
4	P3Fuc	Numeric	8	2		None	None	8	Right	Scale	Input
5	P3Neu5Ac	Numeric	8	2		None	None	8	Right	Scale	Input
6	P3Man	Numeric	8	2		None	None	8	Right	Scale	Input
7	P3Glc	Numeric	8	2		None	None	8	Right	Scale	Input
8	P3GalNAc	Numeric	8	2		None	None	8	Right	Scale	Input
9	P3GlcNac	Numeric	8	2		None	None	8	Right	Scale	Input

3. Choose ‘Analyze’ > ‘Classify’ > ‘Discriminant analysis’ to construct an LDA model.
4. Assign the lectin as the grouping variable, and the fluorescence responses as the independent variables. Define Range as 1 – n, depending on the number of categories.
5. From the ‘Statistics…’ dialogue box, select desired outputs, and from the ‘Classify…’ menu select casewise results, summary table, leave-one-out classification and desired matrices and plots.

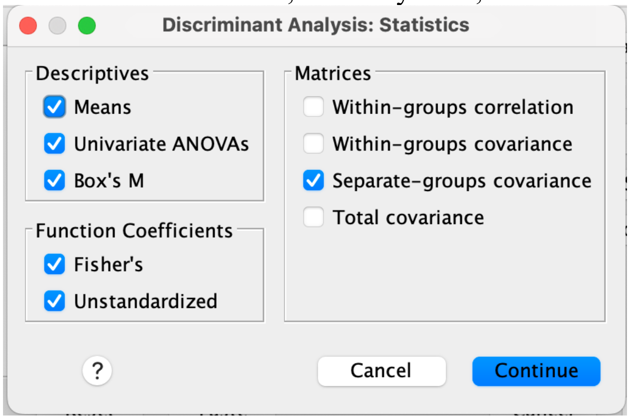

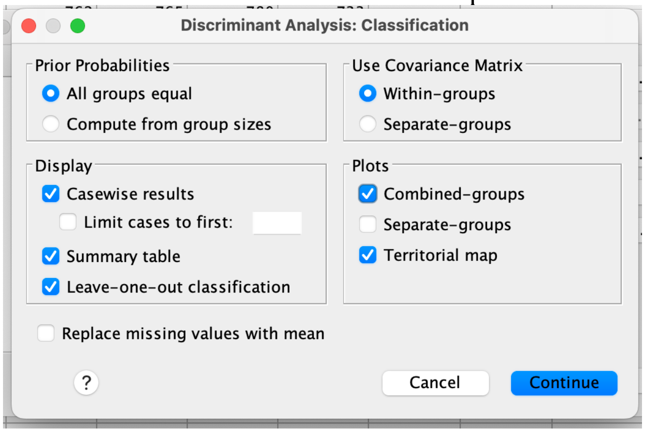

6. If a predictive model is required, chose ‘save’ and select desired outputs, as well as a path for the .xml file.

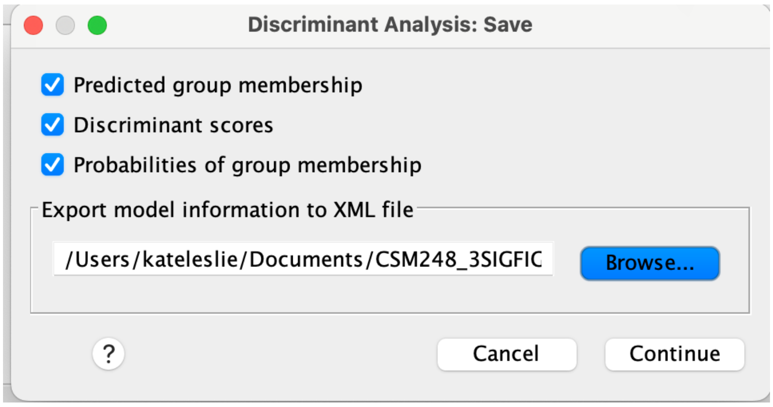

7. Click ‘OK’
